# Supplementary material for: Electronic structure of mononuclear and radical-bridged dinuclear cobalt(II) single-molecule magnets
Source: Nat Commun. 2025 Mar 4;16:2157. doi: 10.1038/s41467-025-57210-0 (PMC11880546; doi:10.1038/s41467-025-57210-0)
Supplement: Supplementary file 1 — Supplementary Information [file 41467_2025_57210_MOESM1_ESM.pdf]

Supporting Information for "Electronic structure of mononuclear and radical-bridged dinuclear cobalt(II) single-molecule magnets"

David Hunger[1+], Julia Netz[2+], Simon Suhr[3+], Komalavalli Thirunavukkuarasu[4], Hans Engelkamp[5], Björn Fåk[6], Uta Albold[7], Julia Beerhues[3], Wolfgang Frey[8], Ingo Hartenbach[3], Michael Schulze[9], Wolfgang Wernsdorfer[9], Biprajit Sarkar[3,7*], Andreas Köhn[2*], and Joris van Slageren[1*]

[1]Institute of Physical Chemistry, University of Stuttgart, Pfaffenwaldring 55, 70569 Stuttgart, Germany
[+]Contributed equally to this work
[2]Institute of Theoretical Chemistry, University of Stuttgart, Pfaffenwaldring 55, 70569 Stuttgart, Germany
[3]Institute of Inorganic Chemistry, University of Stuttgart, Pfaffenwaldring 55, 70569 Stuttgart, Germany
[4]Department of Physics, Florida A&M University, Tallahassee, Florida 32307, United States
[5]HFML-FELIX, Toernooiveld 7, 6525 ED Nijmegen, The Netherlands
[6]Insitut Laue-Langevin, 71 Avenue des Martyrs, 38000 Grenoble, France
[7]Institute of Chemistry and Biochemistry, Freie Universität Berlin, Fabeckstraße 34-36, 14195 Berlin, Germany
[8]Institute of Organic Chemistry, University of Stuttgart, Pfaffenwaldring 55, 70569 Stuttgart, Germany
[9]Physikalisches Institut, Karlsruhe Institute of Technology, Wolfgang-Gaede-Straße 1, 76131 Karlsruhe, Germany
[*]Corresponding authors, e-mail: biprajit.sarkar@iac.uni-stuttgart.de, koehn@theochem.uni-stuttgart.de, slageren@ipc.uni-stuttgart.de

Contents

S1 Supplementary Note 1 - Characterization and Structure **2**
S1.1 Analytical Data . . . 2
S1.2 Crystallographic Data . . . 3
S2 Supplementary Note 2 - Magnetization Curves **6**
S3 Supplementary Note 3 - Dynamic of the Magnetization **7**
S4 Supplementary Note 4 - Far-Infared and Raman spectroscopy **17**
S4.1 Compound 1 . . . 18
S4.2 Compound 2 . . . 22
S5 Supplementary Note 5 - Inelastic neutron scattering **25**
S6 Supplementary Note 6 - Computed electronic structure **29**
S6.1 Active space for the compound 2 . . . 29
S6.2 Sample Molpro input file . . . 29
S6.3 Exchange coupling and spin ladders in compound 2 . . . 31
S6.4 Influence of zero-field splitting and exchange coupling . . . 32
S7 Supplementary Note 7 - Spin-phonon coupling simulations **34**
S7.1 Model simulations for the mononuclear case . . . 34
S7.2 Numerical determination of spin-phonon coupling strength . . . 34
S7.3 Additional simulations for the mononuclear case . . . 37
S7.4 Consideration of phonon polarization . . . 37
S7.5 Simulations for the dinuclear case . . . 38
References **39**

S1 Supplementary Note 1 - Characterization and Structure

S1.1 Analytical Data

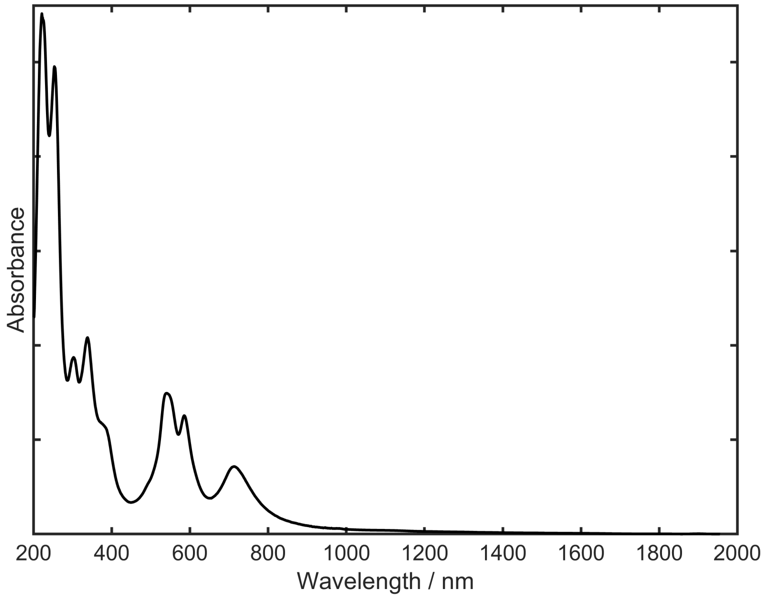

Figure S1. UV/Vis/NIR measurements of an acetonitrile solution of **2**.

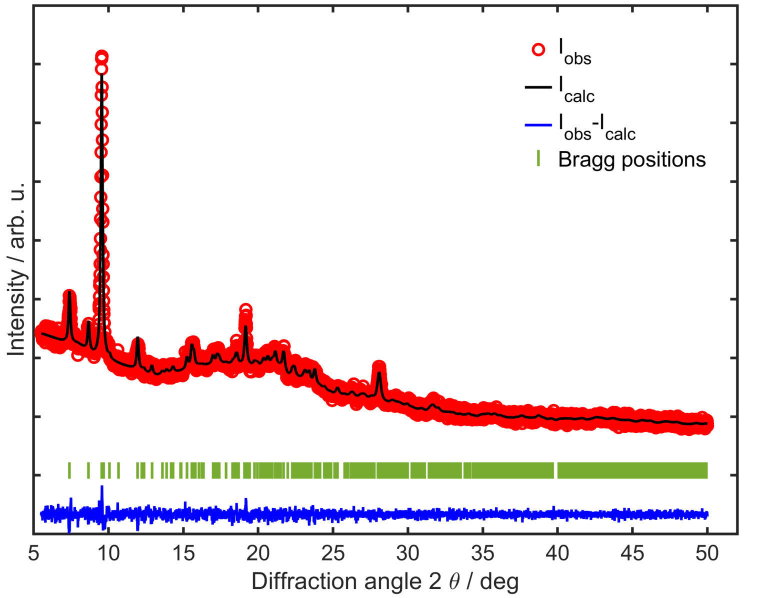

Figure S2. Powder XRD measurement of a batch sample of **2**

Powder XRD was used to prove the phase purity of the batch samples of **2**. The observed reflexes were compared to the calculated positions, that were derived from the single crystal measurement.

S1.2 Crystallographic Data

Table S1. Crystallographic details of **2**

Chemical Formula	$C_{50}H_{90}Co_2K_3N_8O_{32}S_8$
M_r	1806.96
Crystal system	triclinic
Space group	$P\bar{1}$
a (Å)	10.2406(8)
b (Å)	13.0127(11)
c (Å)	17.1988(13)
α(°)	89.956(4)
β(°)	74.191(4)
γ(°)	68.523(3)
V (Å^3)	2039.5(3)
Z	1
Density ($g \cdot cm^{-1}$)	1.471
F(000)	941
Radiation type	MoKα
μ(mm^1)	0.846
Crystal size	0.262 x 0.177 x 0.147
Meas. Refl.	35245
Indep. Refl.	8290
Observ. [$I > 2\sigma(I)$] refl.	6489
R_{int}	0.0279
R [$F^2 > \sigma(F^2)$], wR(F^2), S	0.0594, 0.1489, 1.047
$\Delta\rho$max, $\Delta\rho$min (eÅ^{-3})	1.273, 1.066
CCDC code	2193374

CheckCif flags a B-level alert for this structure: PLAT420_ALERT_2_B D-H Bond Without Acceptor O80-H80B
Author Response: There is a chain of 3 H_2O from sulfonamide, where one water molecule is H-bound to two sulfonamides to the K-coordinated H_2O. The three H_2O molecules could not be reasonably modeled and where removed using "Squeeze" (A. Spek). The highly disordered 3rd water in the chain also is located near a special postion at the cell edge.

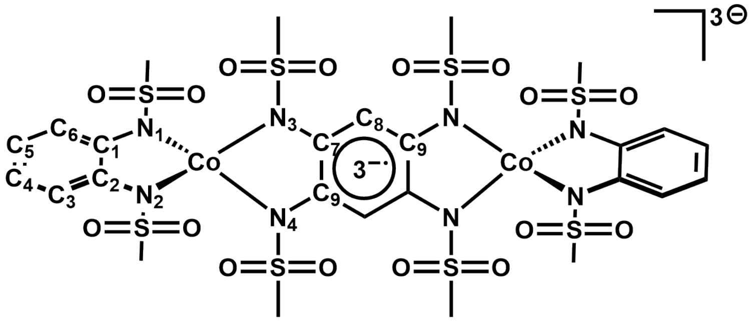

Figure S3. Numbering of the atoms in the dinuclear compound **2**.

Table S2. Experimentally found distances in the crystal structure of **2**.

	Bond length [Å]
Co1 – N1	1.975(3)
Co1 – N2	1.984(3)
Co1 – N3	1.999(3)
Co1 – N4	2.005(3)
N1 – C1	1.406(5)
N2 – C2	1.414(6)
N3 – C7	1.371(5)
N4 – C9	1.378(5)
C7 – C8	1.390(5)
C7 – C9	1.457(5)
C8 – C9	1.389(5)

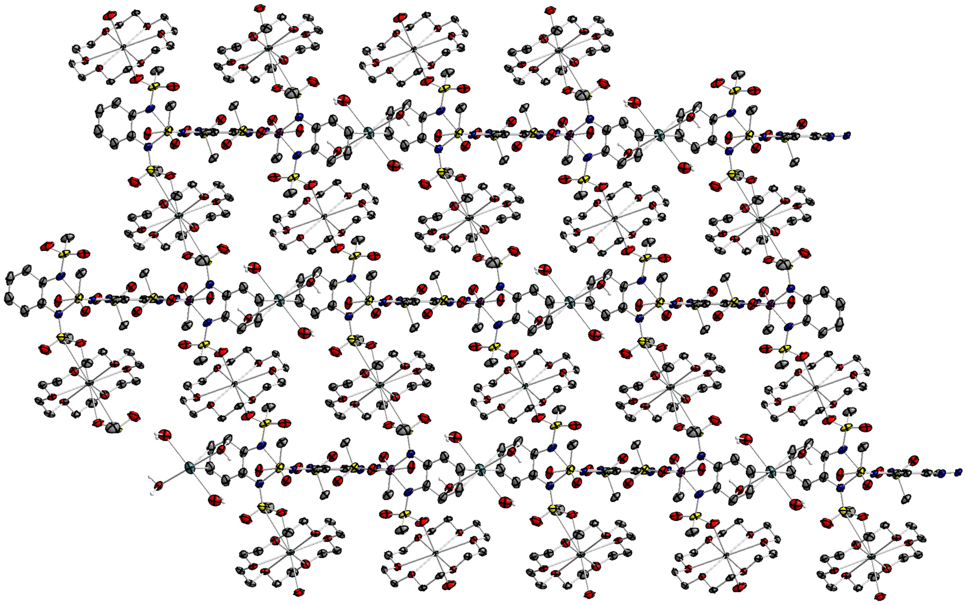

Figure S4. Extended structure of the dinuclear compound **2**

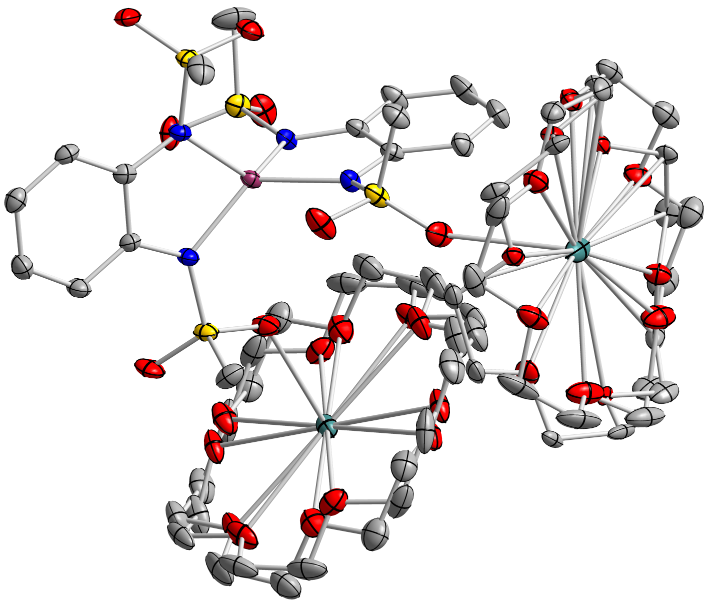

Figure S5. ORTEP-style illustration of the structure of compound **1**[3]. Both crown ether molecules are disordered over two positions. Ellipsoids are drawn at 50% probability. H atoms omitted for clarity

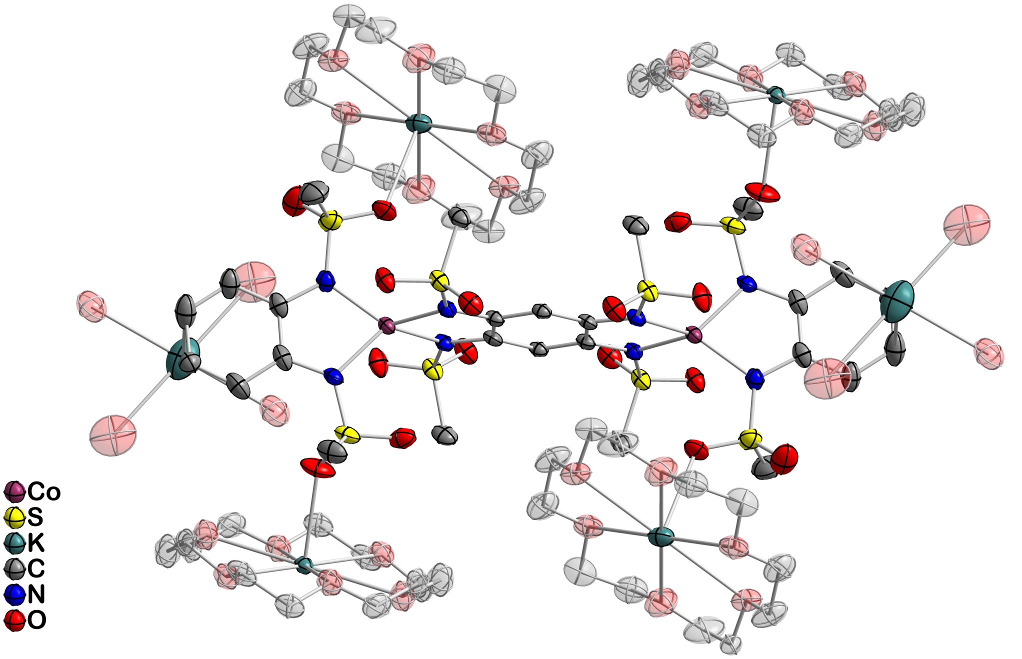

Figure S6. ORTEP-style illustration of the structure of the dimer **2**. Ellipsoids are drawn at 50% probability. H atoms are omitted for clarity.

S2 Supplementary Note 2 - Magnetization Curves

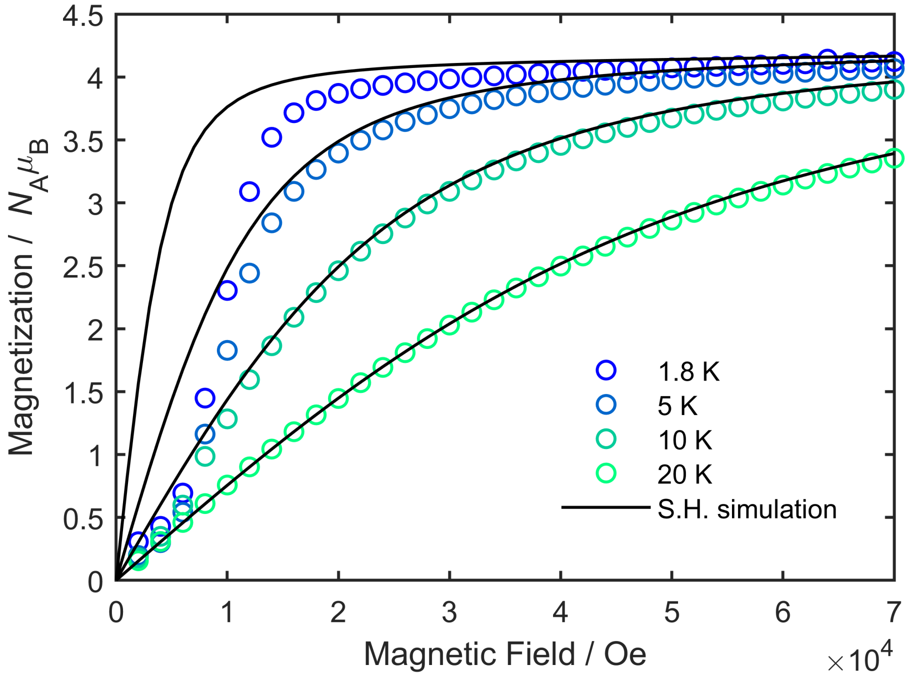

Figure S7. Magnetization measurements of **2** at the indicated temperatures. Measurements are shown as open circles, while spin Hamiltonian simulations based on the parameter set that was obtained from the χT measurement are shown as black solid lines

S3 Supplementary Note 3 - Dynamic of the Magnetization

In order to explore the magnetization dynamics of **2** and to compare them to the already known dynamics of **1**[1] as well as to the structurally related dimer that was published in 2019[2], ac susceptibility was measured at temperatures from 1.8 to 25 K in zero external magnetic field as well as in applied fields. The measurements were carried out on a pressed pellet of **2** with a sample mass of 4.5 mg. For **2**, a clear out-of-phase signal is observed in the whole temperature regime at zero field. At 1.8 K, a maximum in χ'' is observed at a frequency of 0.13 Hz. This maximum shifts towards higher frequencies with an increase in temperature to 42 Hz at 25 K. In order to extract the relaxation times of the magnetization, the in-phase and out-of-phase component of the dynamic susceptibility was fitted in analogy to the procedure used in 2 by means of modified Debye functions. A direct comparison of the three compounds is shown in the main text. Here, a comparable relaxation behavior of **2** and of the dimer published by Albold et al.[2] (**3**) is found. In this context, both dimers show a drastically increased relaxation time in comparison to the monomer **1**, which highlights the persistence of the relaxation behavior of radical bridged complexes of bmsab towards modifications of the ligand backbone on the one hand and on the other hand the potential of radical bridged structures in the context of molecular magnetism.

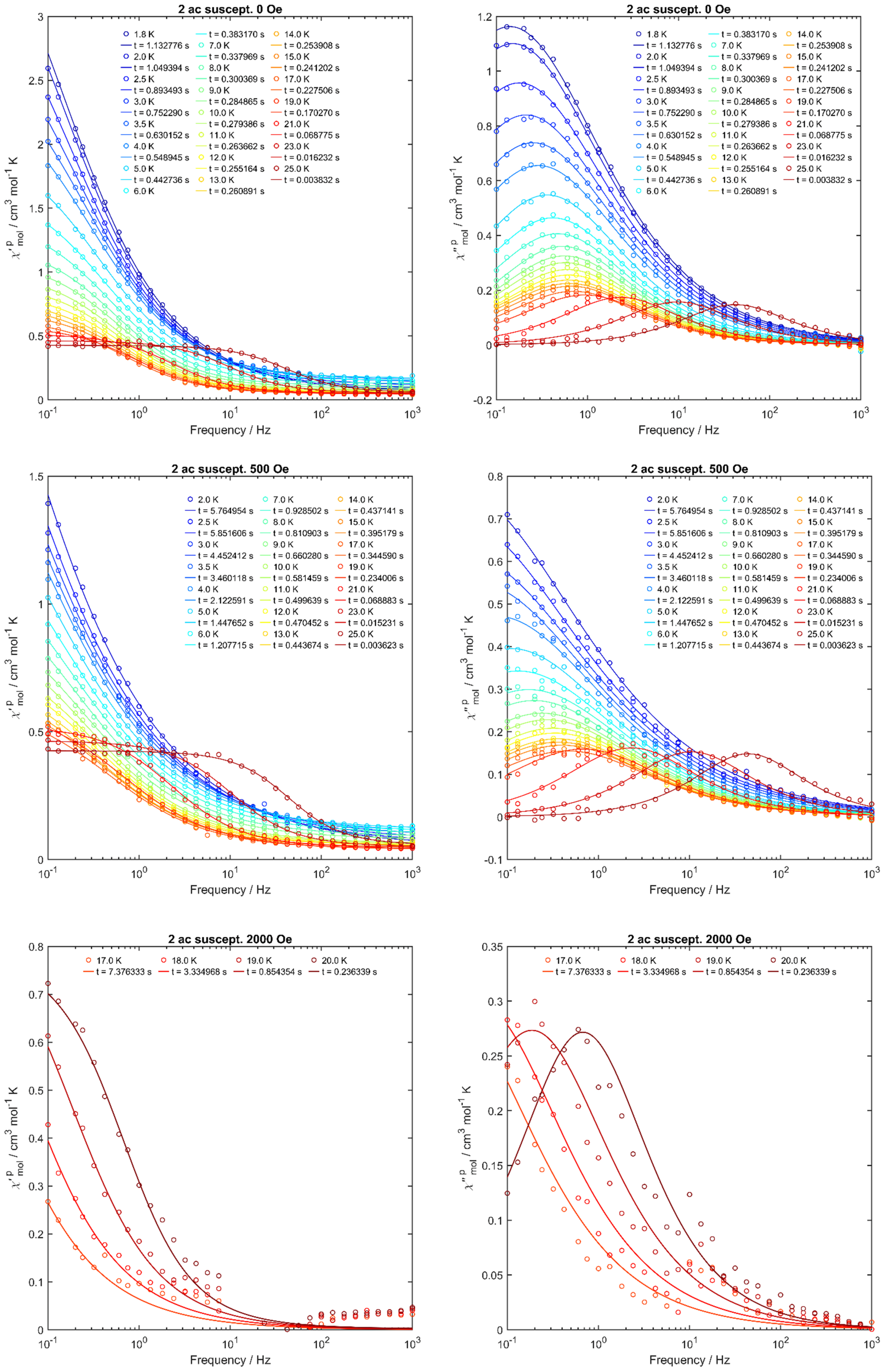

Figure S8. Overview of the various ac measurements of **2** in zero and applied fields. Magnetic fields and temperatures are indicated in the figures.

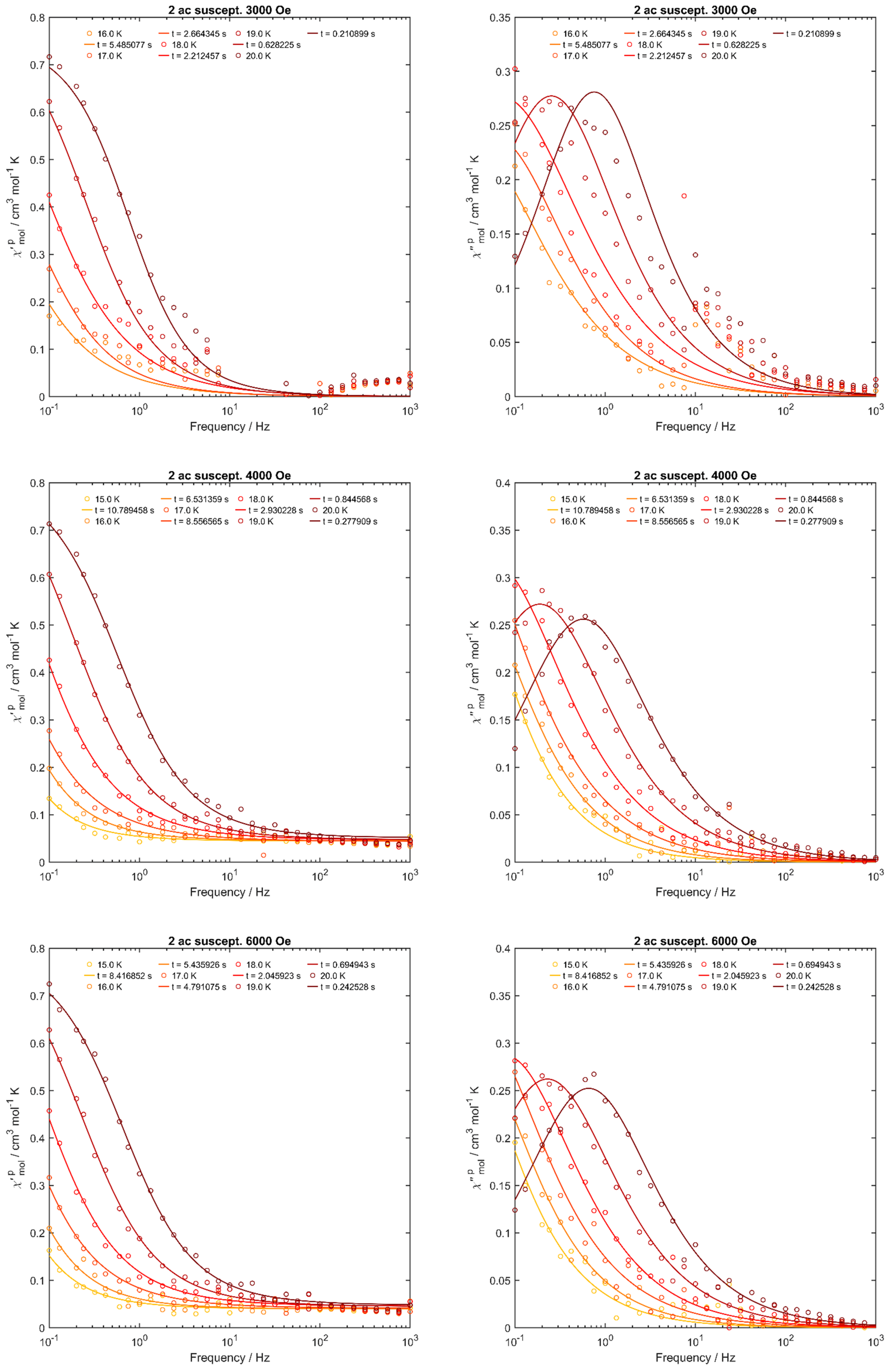

Figure S9. Overview of the various ac measurements of **2** in higher applied fields. Magnetic fields and temperatures are indicated in the figures.

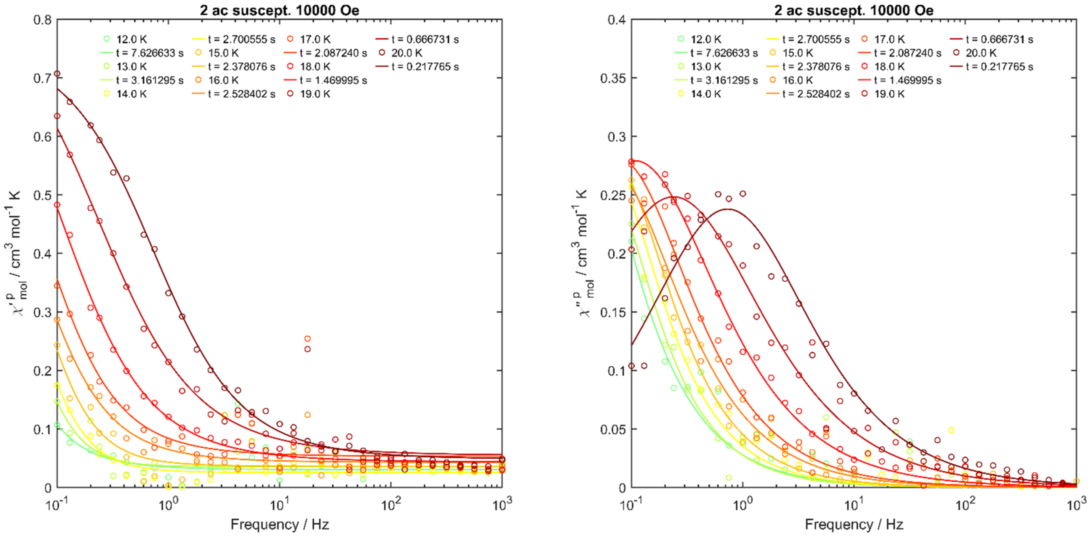

Figure S10. Overview of the ac measurements of **2** at 10000 Oe. Temperatures are indicated in the figure.

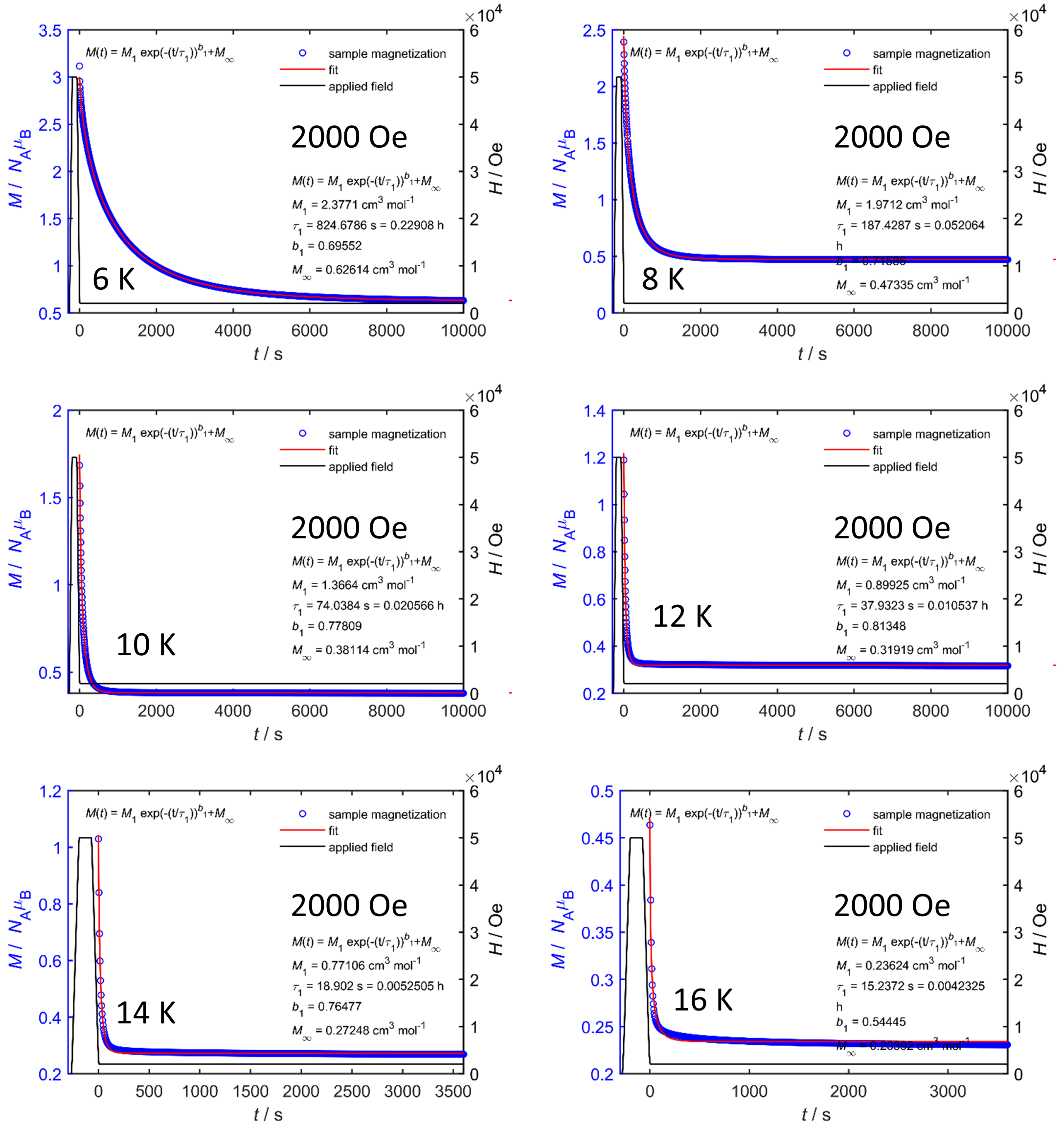

Figure S11. Overview of the dc relaxation measurements of **2** at 2000 Oe. Temperatures are indicated in the figure. Note that the left y-axis is not always the same scale as the right one.

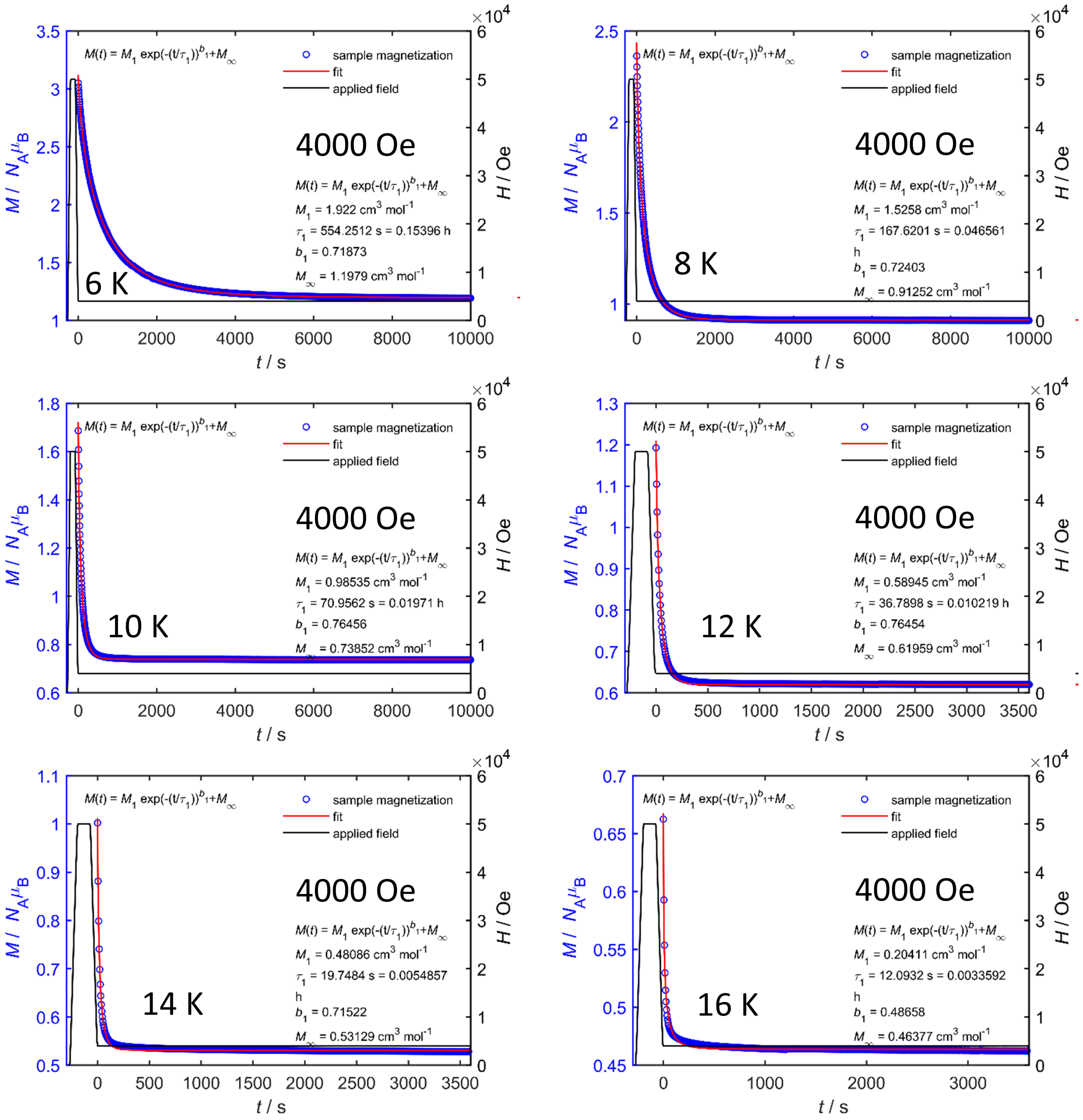

Figure S12. Overview of the dc relaxation measurements of **2** at 4000 Oe. Temperatures are indicated in the figure. As above, both y-axis are scaled differently.

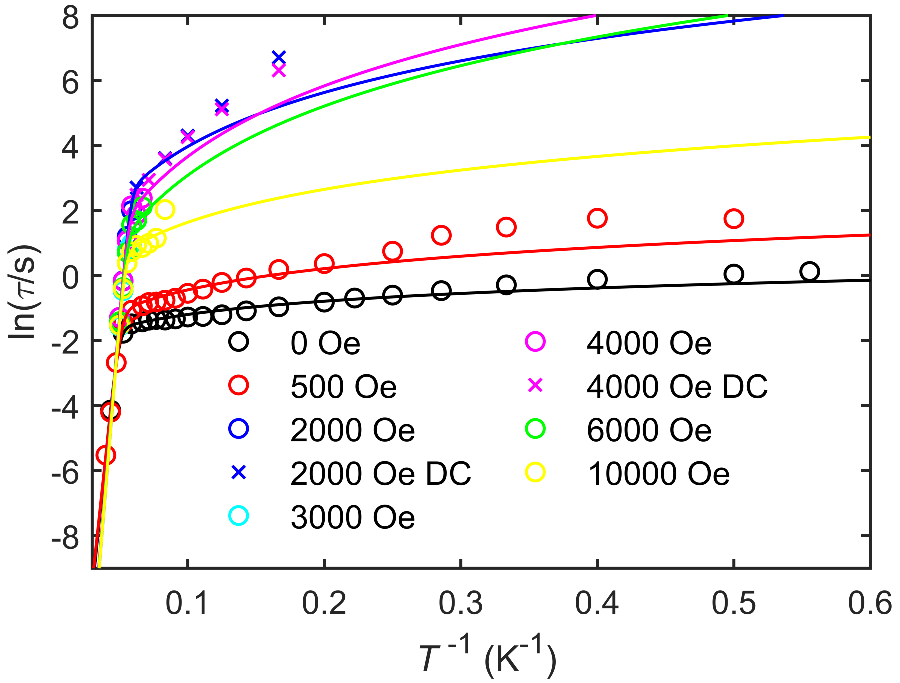

Figure S13. Natural logarithm of the different relaxation times of **2** in applied fields as a function of the inverse temperature. The applied external fields are indicated in the figure. Measured values are shown as symbols while fits based on the equation given in the main text are shown as solid lines in the corresponding color. Fit parameters are found below.

Table S3. Fit parameters of the fits to the natural logarithm of the relaxation times of **2** as a function of the inverse temperature. The corresponding function is found in the main text.

	500 Oe	2000 Oe	4000 Oe	6000 Oe	10000 Oe
τ_0/s	$6.1(9)\cdot 10^{-10}$	$2.2(7)\cdot 10^{-12}$	$2.6(8)\cdot 10^{-12}$	$2.4(7)\cdot 10^{-12}$	$2(1)\cdot 10^{-12}$
$U_{\mathrm{eff}}/\mathrm{cm}^{-1}$	273(1)	353(4)	353(20)	353(46)	353(22)
$C/\mathrm{s}^{-1}\mathrm{K}^{-n}$	0.177(5)	$7.5(4)\cdot 10^{-5}$	$1.9(8)\cdot 10^{-5}$	$3.8(4)\cdot 10^{-5}$	0.007(5)
n	0.94(11)	2.4(7)	3.1(9)	3.0(4)	1.46(60)

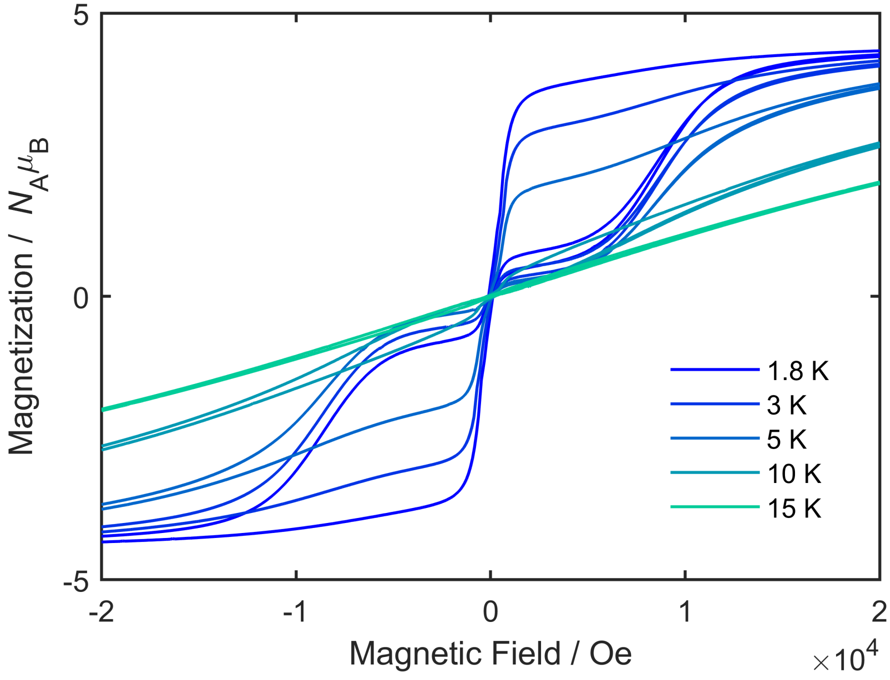

Figure S14. Hysteresis measurements of **2** at the indicated temperatures.

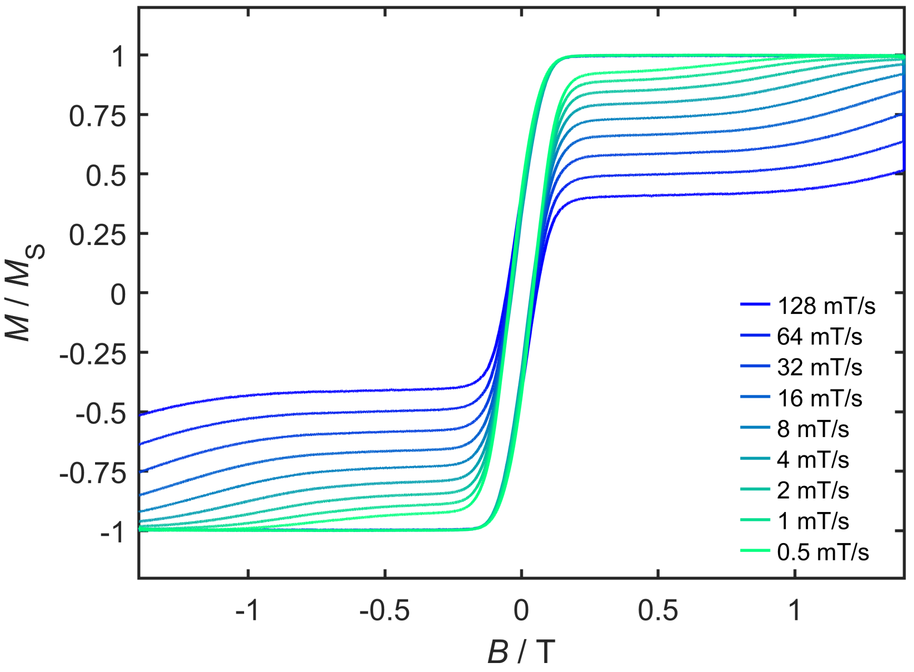

Figure S15. microSQUID measurements of **2** at a temperature of 30 mK at the indicated sweeping speeds. Magnetization values are normalized to the saturation magnetization.

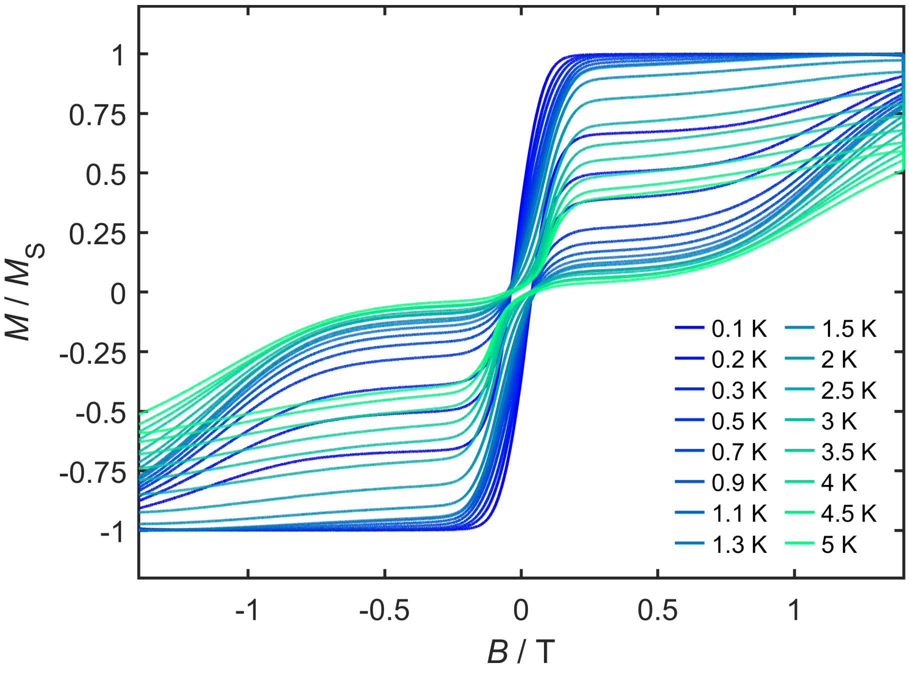

Figure S16. Temperature dependent microSQUID measurements of **2** at a constant sweeping speed of 8 mT/s. Again, the magnetization values are normalized to the saturation magnetization.

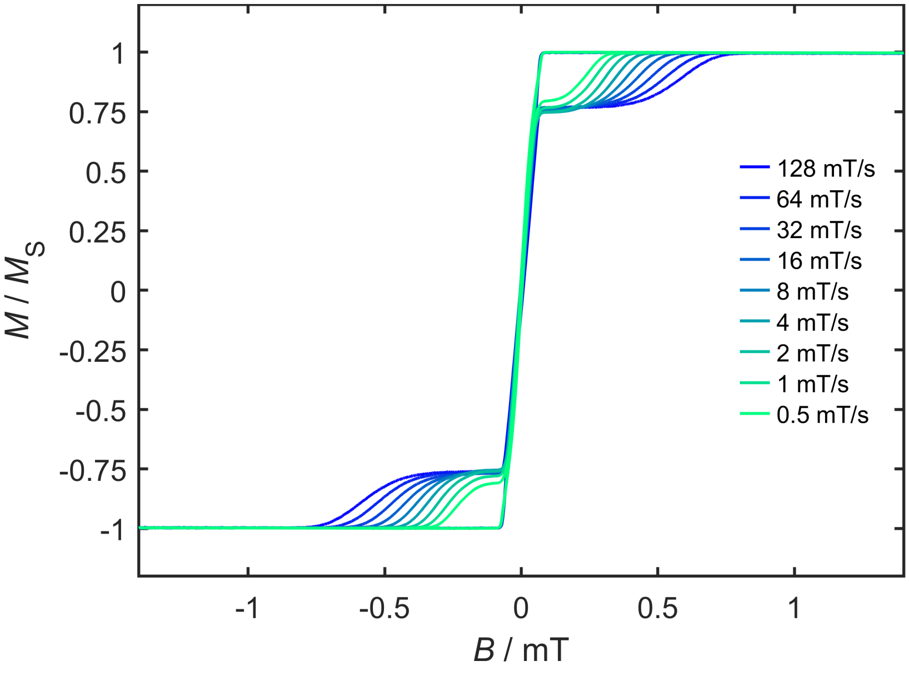

Figure S17. microSQUID measurements of **1** at a temperature of 30 mK at the indicated sweeping speeds. As for **2**, the magnetization values are normalized to the saturation magnetization.

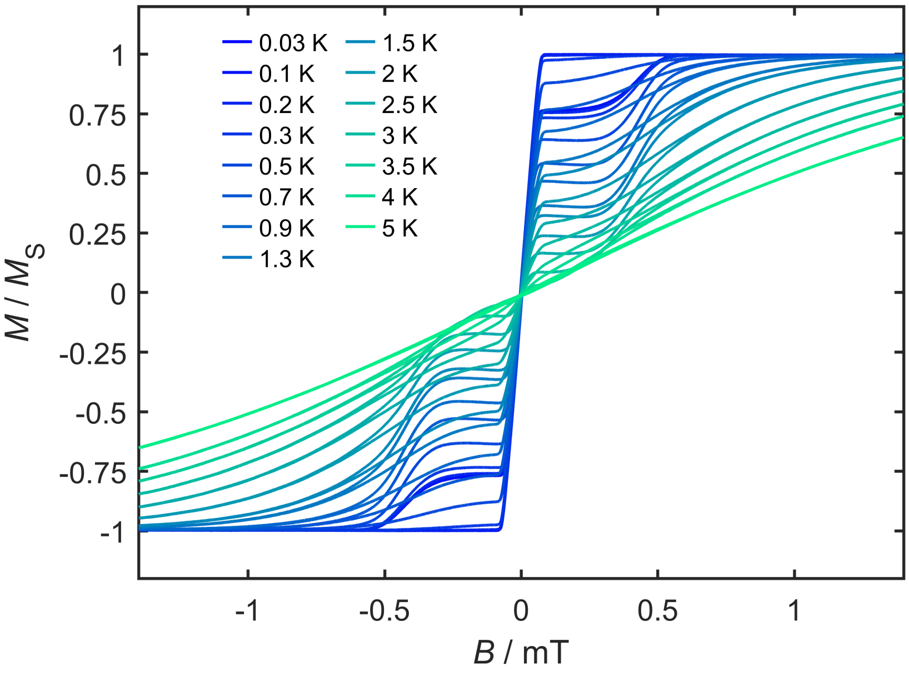

Figure S18. Temperature dependent microSQUID measurements of **1** at a constant sweeping speed of 16 mT/s. As above, the magnetization values are normalized with respect to the saturation magnetization.

S4 Supplementary Note 4 - Far-Infared and Raman spectroscopy

The Far-Infrared (FIR) spectra are measured as transmission spectra $Tr(\nu,B)$ for a static magnetic field B. The transmission can be related to the molar absorbance $\varepsilon(\nu,B)$ of the system by the integrated Lambert-Beer law:

$$Tr(\nu,B) = \frac{I(\nu,B)}{I_{\text{ref}}(\nu)} = \exp\left(-c_{\text{spec}}\,\varepsilon(\nu,B)\right). \tag{S1}$$

In this equation, $I(\nu,B)$ is the actual measured signal, $I_{\text{ref}}(\nu)$ is the reference signal without sample and c_{spec} a constant that depends on the concentration of the absorbing species and other measurement-specific factors. In the main text we will only consider normalized spectra computed as the ratio $I(\nu,0)/I(\nu,B)$. Inserting Eq. S1 leads to

$$\frac{Tr(\nu,0)}{Tr(\nu,B)} = \frac{I(\nu,0)}{I(\nu,B)} = \exp\left(c_{\text{spec}}(\varepsilon(\nu,B)-\varepsilon(\nu,0))\right) \approx 1 + c_{\text{spec}}(\varepsilon(\nu,B)-\varepsilon(\nu,0)) \tag{S2}$$

which shows that this normalized spectrum is approximately proportional to the difference in absorption of the system with and without applied magnetic field.

S4.1 Compound 1

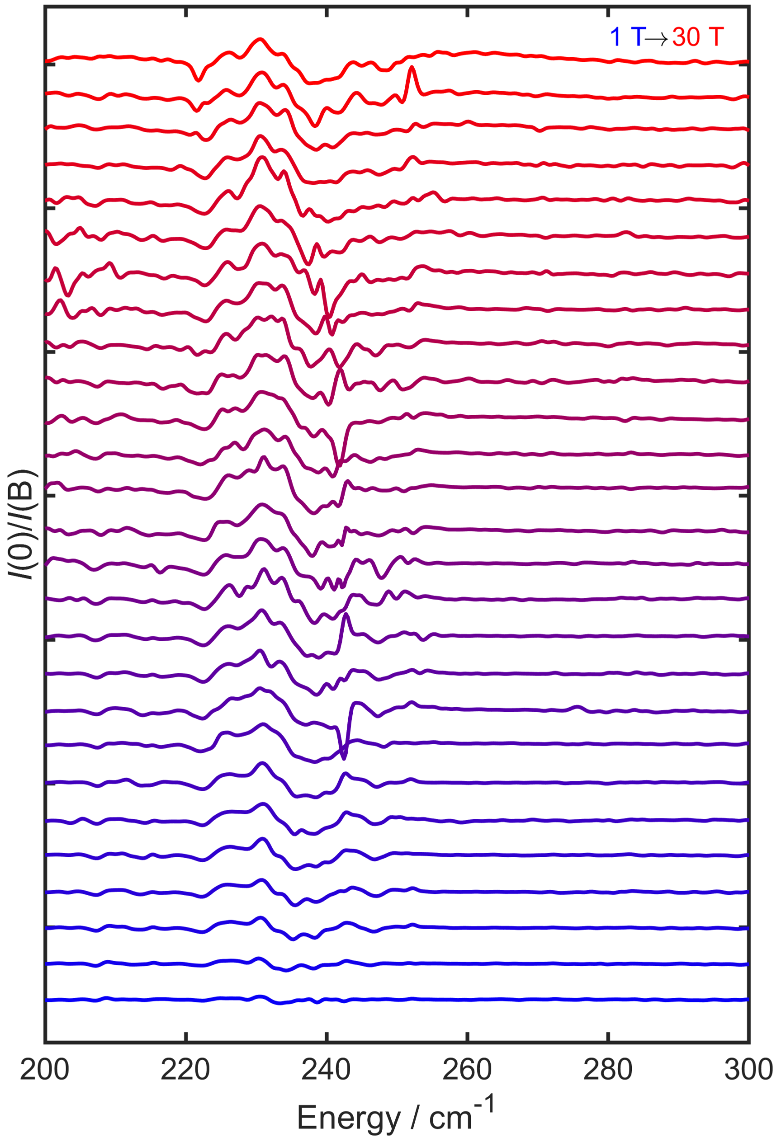

Figure S19. Field dependent FIR spectra on **1** referenced to the 0 T spectrum at the indicated fields at a temperature of 3 K. Measurements were carried out on a 8 mm diameter pressed pellet of 20 percent sample in paraffin. The spectra were measured on a setup at the high magnetic field laboratory (HFML) in Nijmegen (Netherlands), consisting of a Bruker Vertex 80v spectrometer coupled to a 33 T Bitter type magnet.

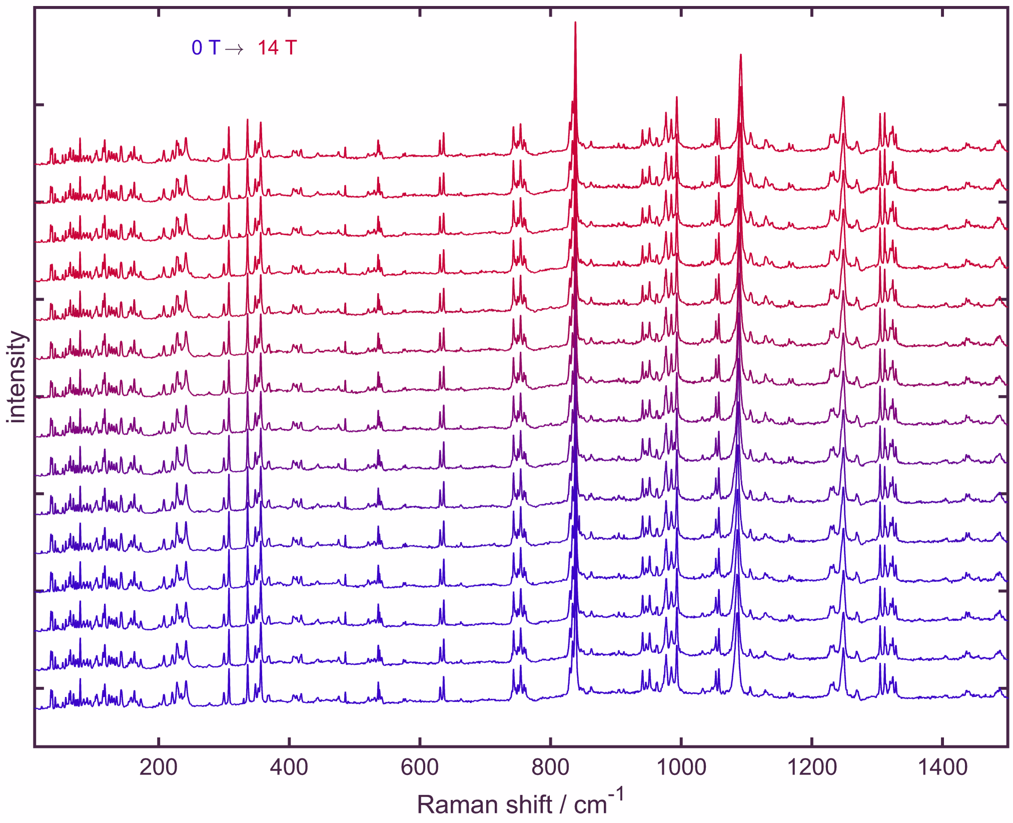

Figure S20. Full Raman spectra for compound **1**. Measured fields are indicated in the figure.

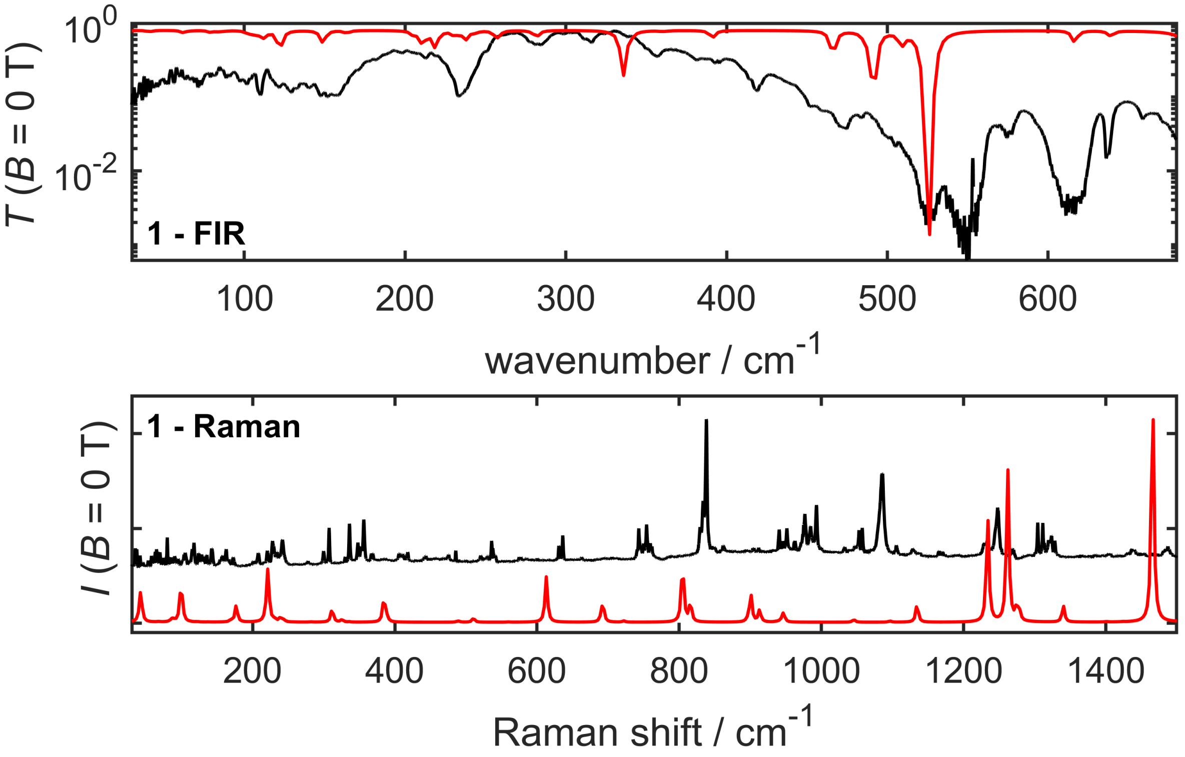

Figure S21. FIR and Raman spectrum comparison for the mononuclear compound **1** in zero field. Measured spectra are shown as black solid lines, calculated spectra as red lines.

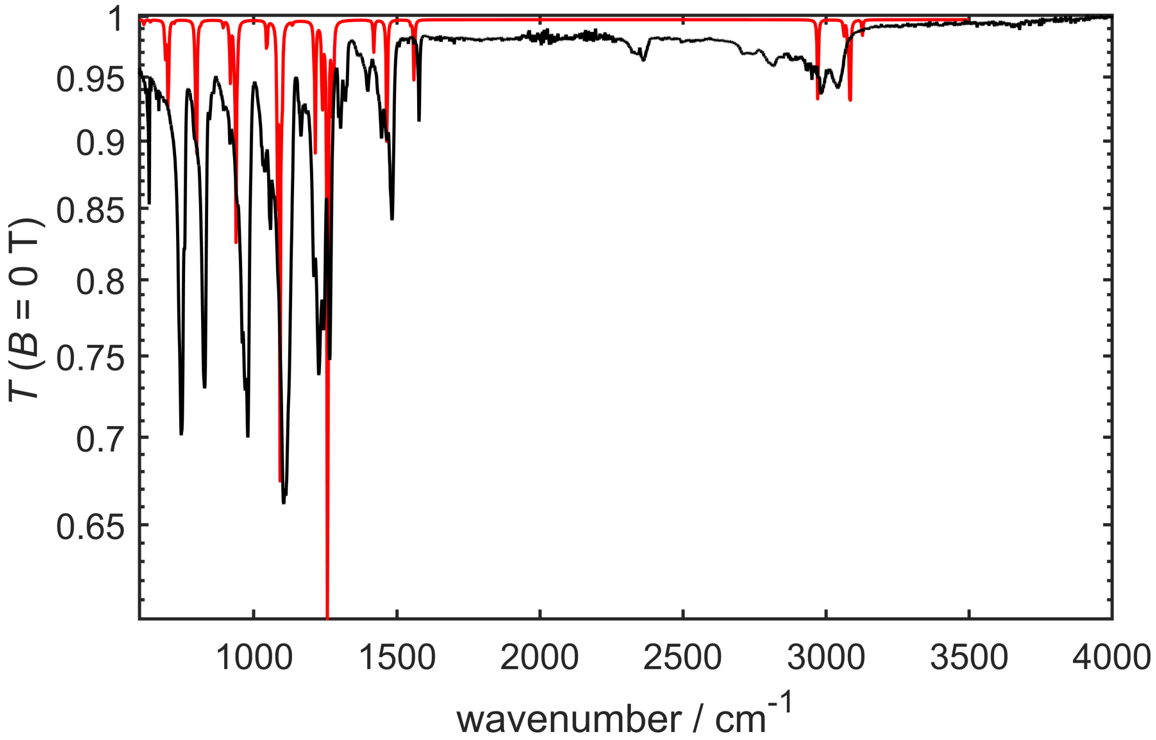

Figure S22. Comparison of the same calculated spectrum (red) as in figure S21, but with an extended measured energy regime (black) of **1**. In contrast to the FIR measurements in figure S21, the measurements presented in this figure were carried out at room temperature in an conventional IR setup (Thermo Scientific Nicolet iS5 ATR-IR).

S4.2 Compound 2

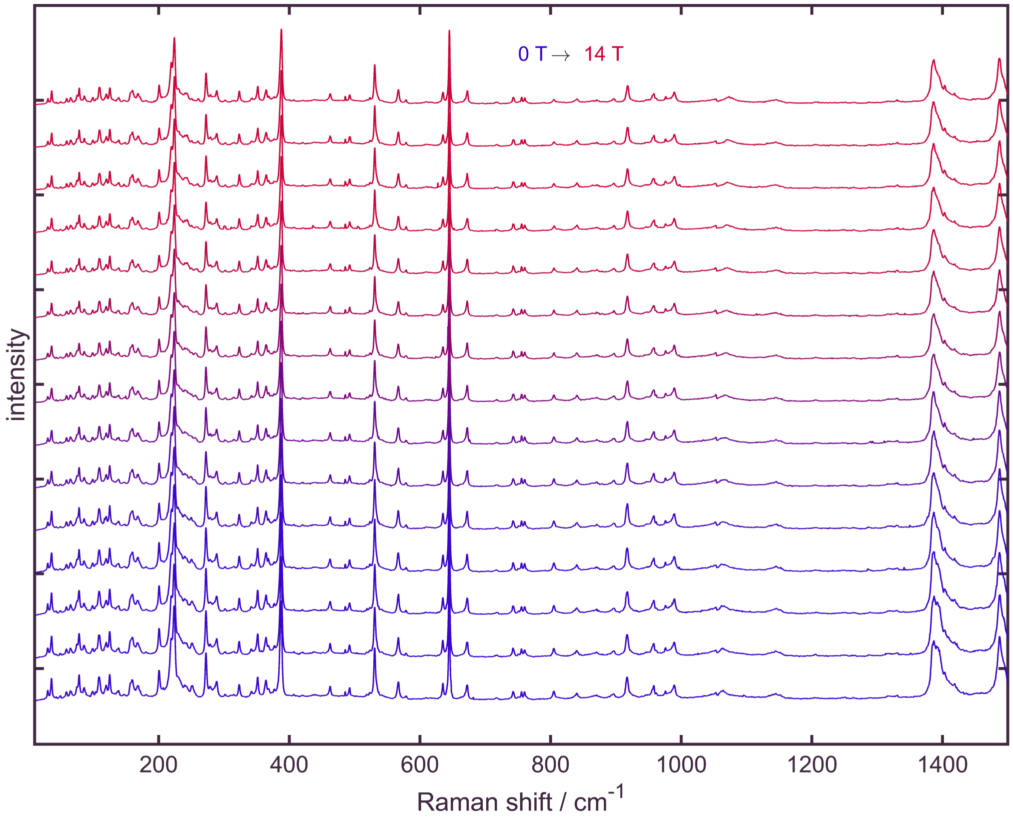

Figure S23. Full Raman spectra for compound **2**. Measured fields are indicated in the figure.

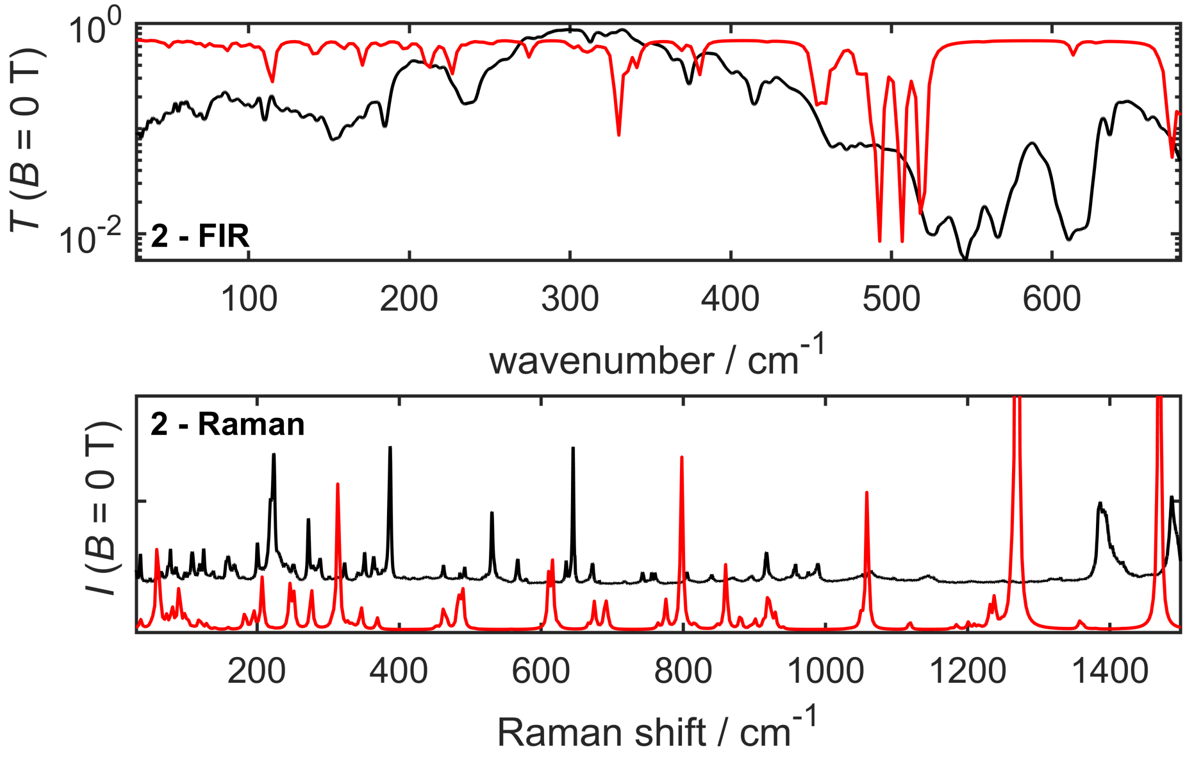

Figure S24. FIR and Raman spectrum comparison for the dinuclear compound **2** in zero applied external magnetic field. Measurements are shown in black while calculated spectra are shown in red.

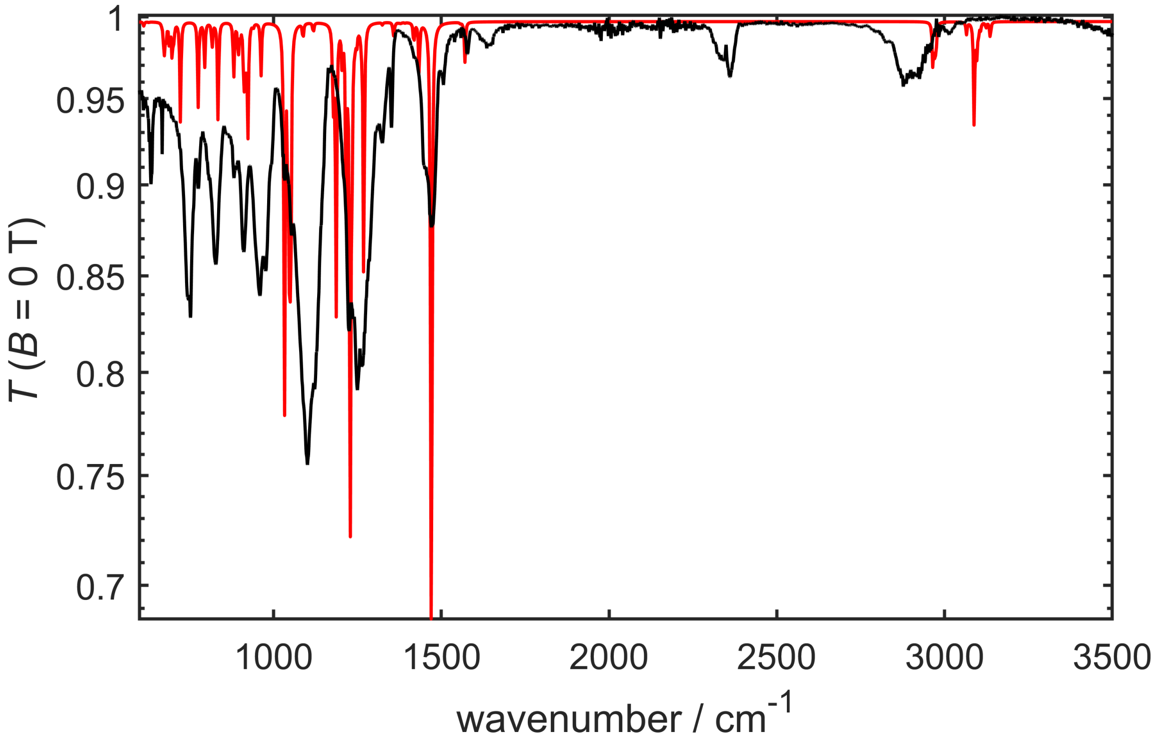

Figure S25. Comparison of the calculated IR spectrum at 0 T (red) of **2** with the corresponding measurement (black). The calculated spectrum is equal to the one shown in figure S24, while the IR spectrum was recorded at room temperature on an conventional instrument (Thermo Scientific Nicolet iS5 ATR-IR).

S5 Supplementary Note 5 - Inelastic neutron scattering

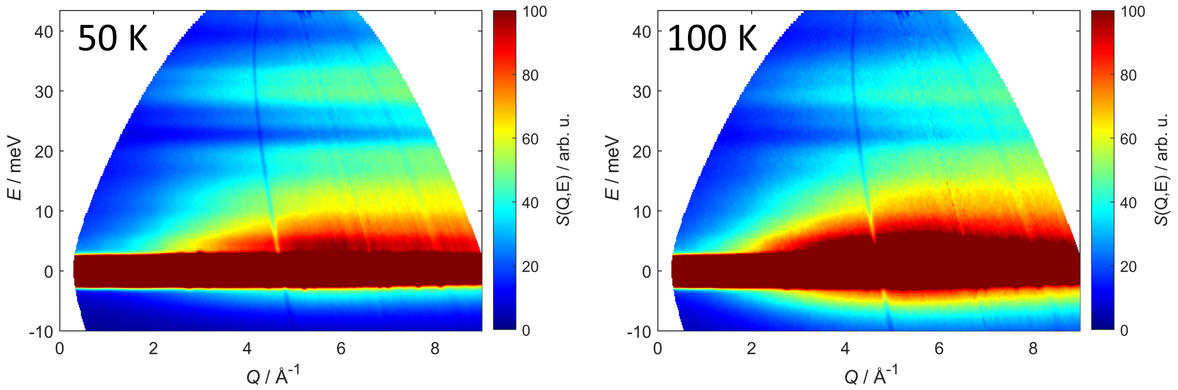

Figure S26. Measured $S(Q,E)$ as a function of the energy transfer E and the momentum transfer Q for **1** at the indicated temperatures. The incident energy was $E_i = 50$ meV.

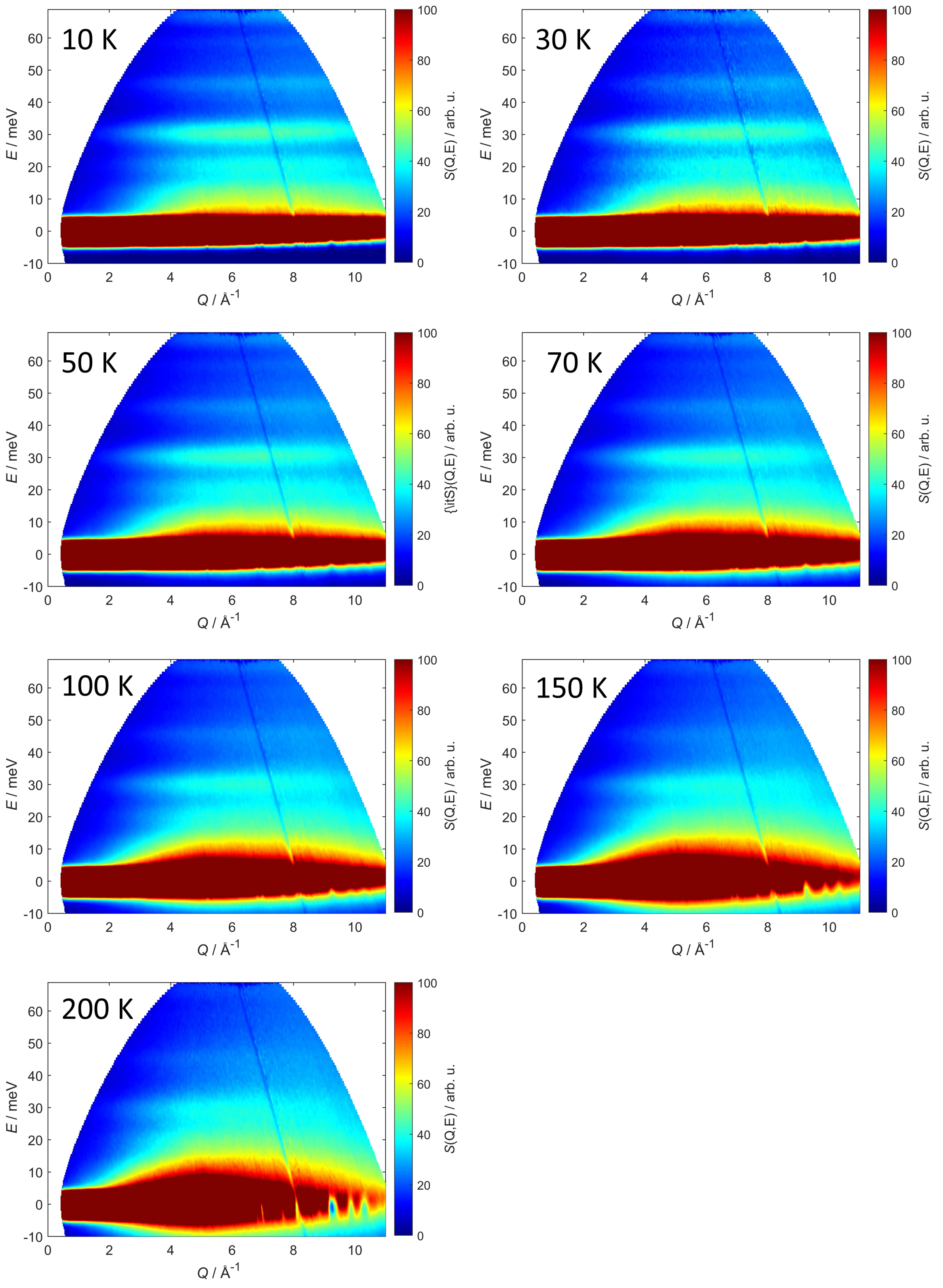

Figure S27. Measured $S(Q,E)$ of **2** at the indicated temperatures. The incident energy was $E_i = 76$ meV.

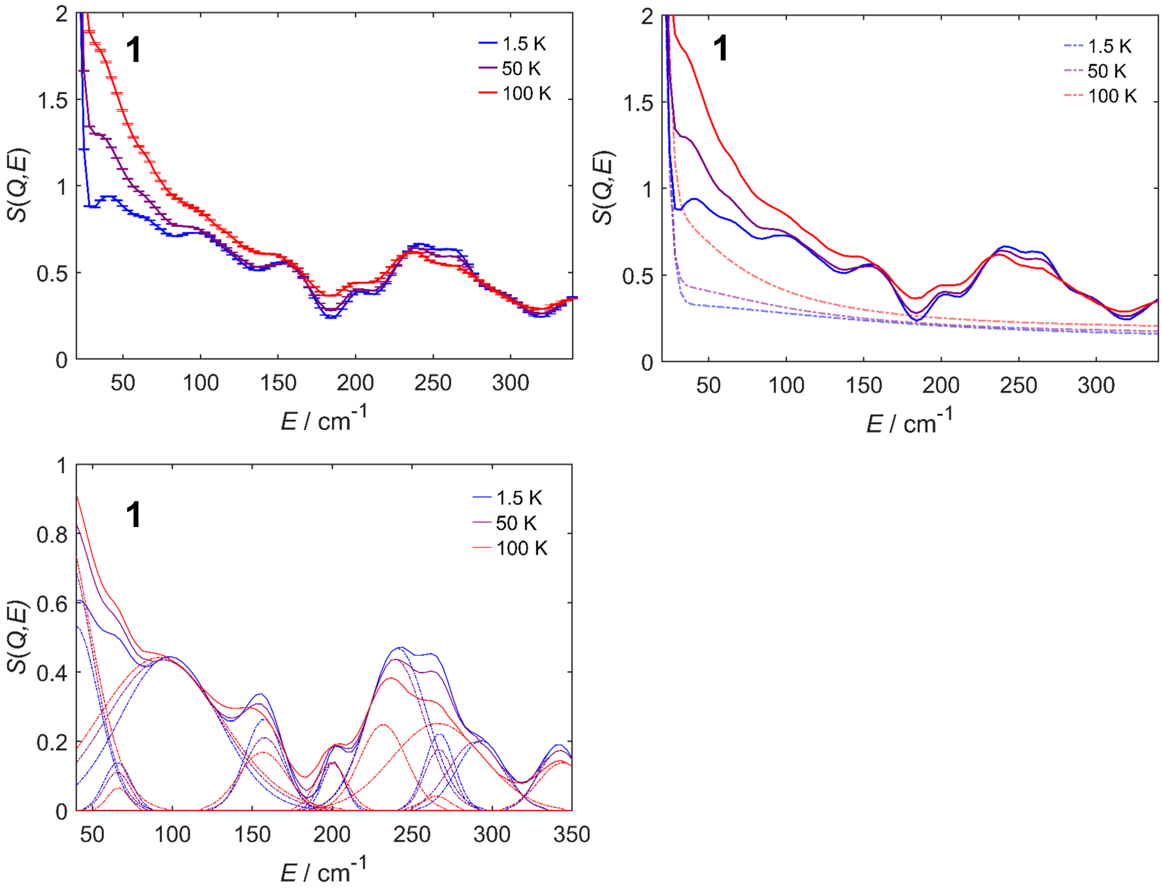

Figure S28. Measured $S(Q,E)$ of **1** at $Q = 3.0 \pm 0.5$ Å^{-1} (top left). Top right: Measured $S(Q,E)$ (solid lines) of **1** and the corresponding Voigtian baselines (dashed), that were subtracted in the analysis. Bottom: Deconvolution of the Gaussian fits, the single Gaussians are shown as dashed lines, the sum of all Gaussians as solid lines.

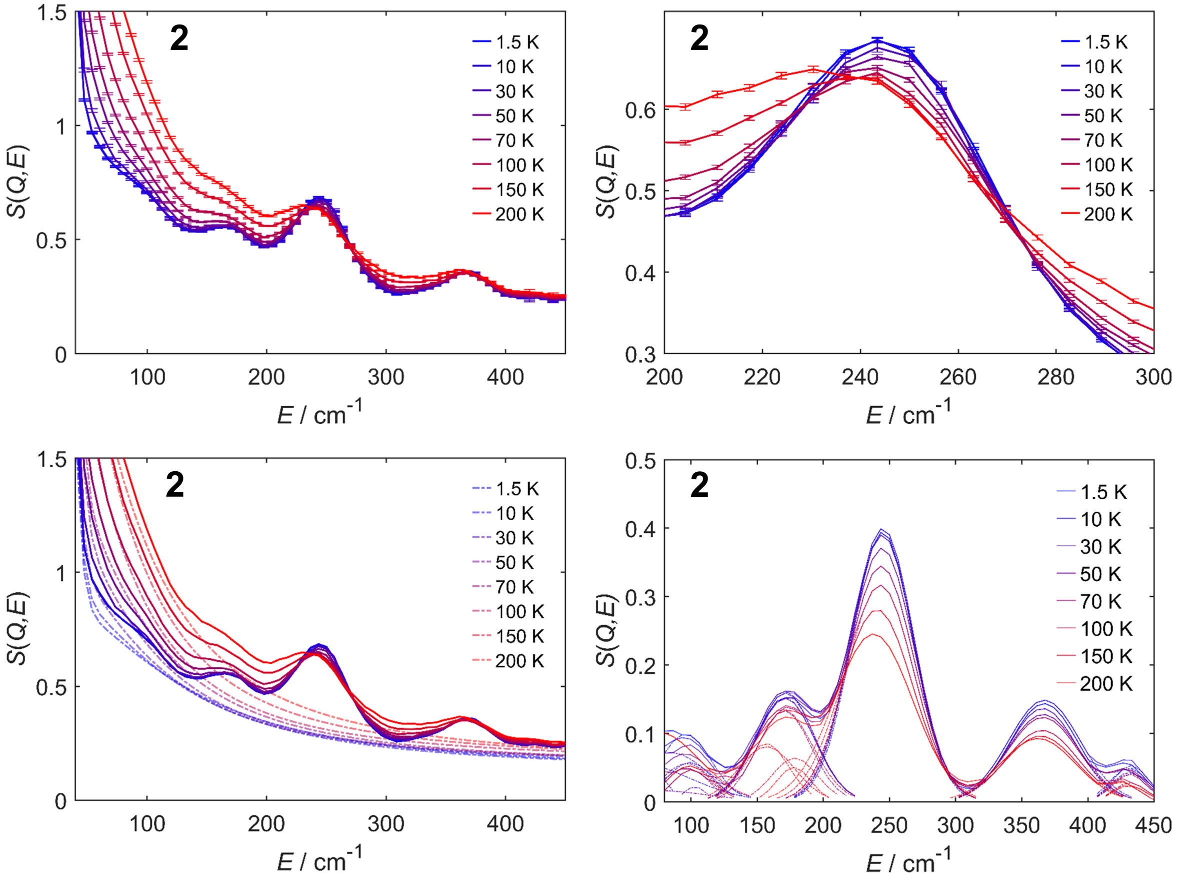

Figure S29. Top left: Measured $S(Q,E)$ of **2** at constant $Q = 3.0 \pm 0.5$ Å^{-1}. Top right: Zoom-in on the region at around 240 cm^{-1}. Bottom left: Measured $S(Q,E)$ of **2** as well as the Voigtian baselines (dashed) that were subtracted in the analysis. Bottom right: Deconvolution of the Gaussian fits. Single Gaussians are shown as dashed lines and the sum of all Gaussians as solid lines.

S6 Supplementary Note 6 - Computed electronic structure

The structures were optimized as described in the main text (see Experimental Section, 'Electronic Structure Calculations').

Computed coordinates of all relevant structures can be found in the Supplementary Data 1. Details of the computed states (spin-orbit coupled states along with their magnetic moments) and the computed IR and Raman spectra of the isolated anions and the normal-mode projected derivatives of the zero-field splitting tensors can be found in the Supplementary Data 2.

S6.1 Active space for the compound 2

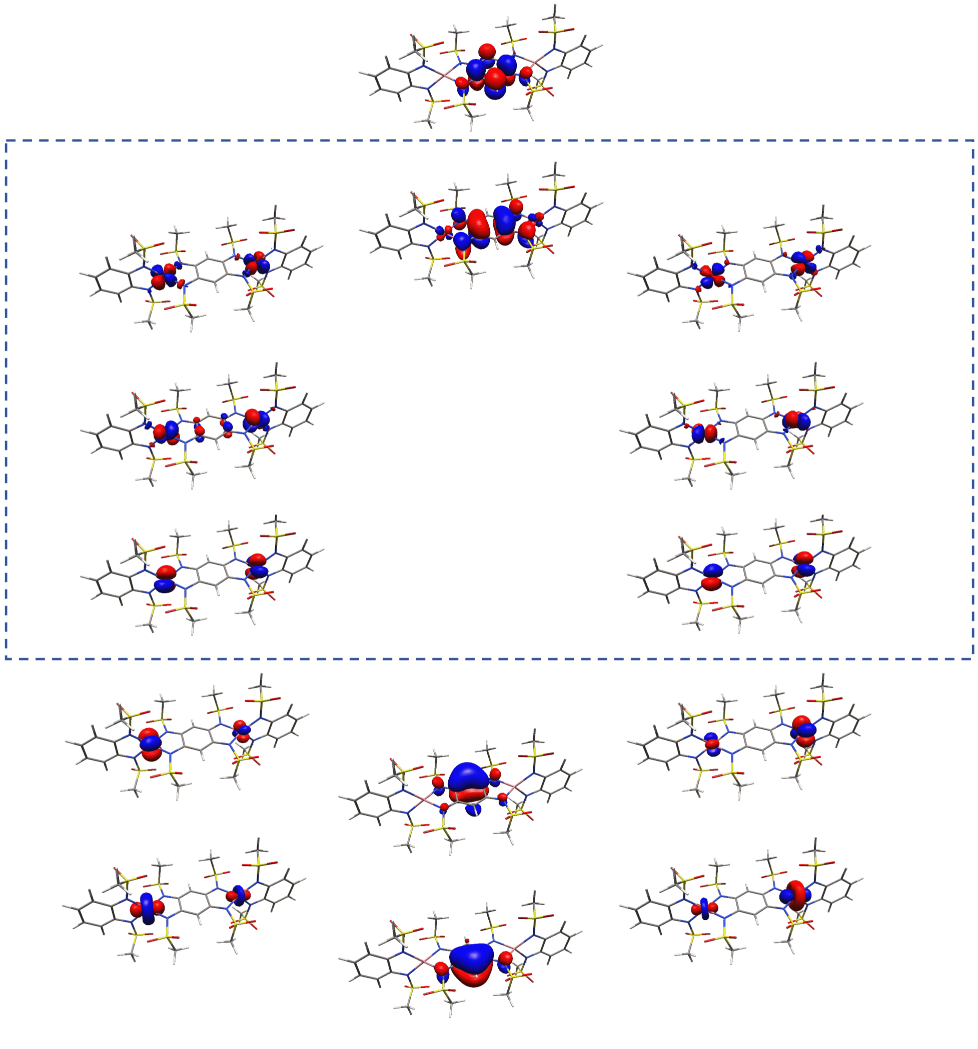

Figure S30. Active orbitals in the CAS(19,14) for compound **2**. The average orbital occupation increases from top to bottom, the seven orbitals collected in the frame are predominantly singly occupied in the lowest-lying spin-ladder states.

S6.2 Sample Molpro input file

Sample input for a computation on **2**:

```
memory,3800,m
gthresh,energy=1e-7
```

```
gprint,orbital,civector
gdirect

ang
nosym
geometry=2-DFT-periodic.xyz
basis={
default=def2-SVP
co=def2-TZVPP
zn=def2-TZVPP
n=def2-TZVPP
}

! initial computation: CAHF for non-oxidized bridge
charge=-4

{avas,thr=0.5,nela=14,locorb=1;
wf,,1,6
center,1,3d
center,2,3d}

{df-multi,cahf
start,2110.2,set=2
cahf,7,274.1,-278.1
cahf,7,279.1,-283.1}

! actual CASSCF computation with radical bridge
charge=-3

{df-multi
occ,284;closed,270
rotate,269.1,271.1
rotate,284.1,294.1
wf,,1,7;state,20
wf,,1,5;state,40
wf,,1,3;state,40
wf,,1,1;state,40}

!if orbital output required:
!for use with gmolpro:
!put,xml
!for use with iboview:
!put,CoRdCo.xml

! redo CAS-CI for smaller number of states
! and save these states for CASPT2 and SO-CI
{df-casci
occ,284;closed,270
wf,,1,7;state,4
wf,,1,5;state,8
wf,,1,3;state,8
wf,,1,1;state,8
save,cirec=5100.2} ! the four sets will be on 5100.2 5200.2 5300.2 5400.2

! PNO-CASPT2 computations
shfpt2=0.45
local,thrpno_occ=1.d-8,thrdist=1.d-6,iext=2
{pno-caspt2,h0=2,coupcor=2,cirec=5100.2,shift=shfpt2,saveheff=5417.2,maxit=100;
wf,,1,7;state,4;thresh,thrdls=1.d-8}
```

```
{pno-caspt2,h0=2,coupcor=2,cirec=5200.2,shift=shfpt2,saveheff=5415.2,maxit=100;
wf,,1,5;state,8;thresh,thrdls=1.d-8}

{pno-caspt2,h0=2,coupcor=2,cirec=5300.2,shift=shfpt2,saveheff=5413.2,maxit=100;
wf,,1,3;state,8;thresh,thrdls=1.d-8}

{pno-caspt2,h0=2,coupcor=2,cirec=5400.2,shift=shfpt2,saveheff=5411.2,maxit=100;
wf,,1,1;state,8;thresh,thrdls=1.d-8}

! Finally: do the spin-orbit CI
! SOC is computed from CASCI states, matrix is updated by effective H from CASPT2
! compute LOP with respect to inversion center
! use HLS=1 to get output for matrix elements (for postprocessing)
{ci;core,270
hlsmat,als,5100.2,5200.2,5300.2,5400.2,heff,5417.2,5415.2,5413.2,5411.2;
expec,lop,,0.,0.,0.
print,HLS=1,VLS=0}
```

S6.3 Exchange coupling and spin ladders in compound 2

The dinuclear complex **2** features a strong exchange coupling to the radical bridge. To improve physical understanding, it is instructive to analyze the electronic spectrum in the absence of spin–orbit coupling. The resulting energy levels are shown in Fig. S31, sorted according to the multiplicity of the states.

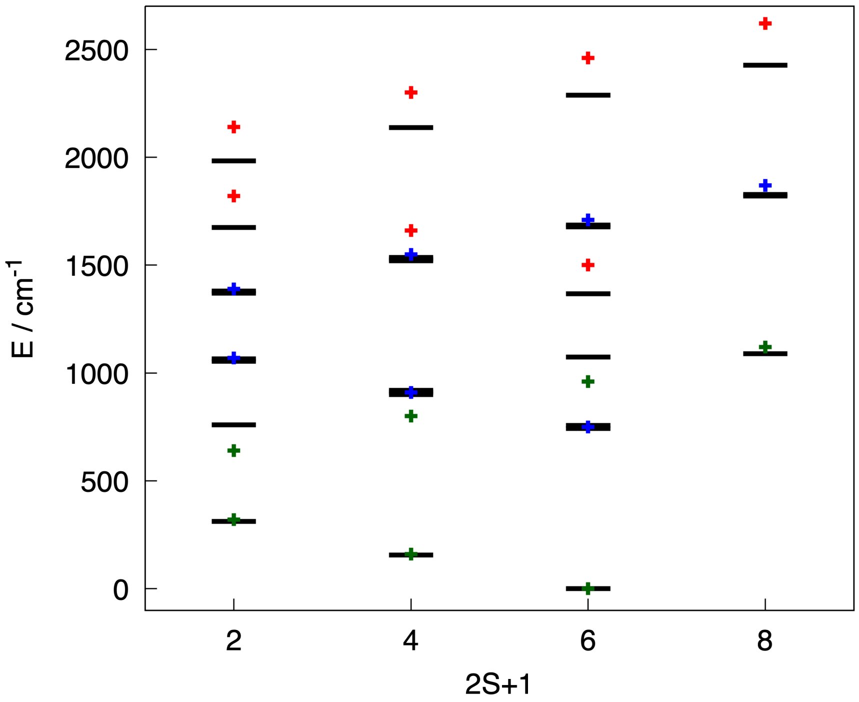

Figure S31. Computed lowest electronic states (horizontal bars) of **2** without spin–orbit coupling, sorted according to their multiplicity. The colored crosses indicate the spin ladders arising from a spin Hamiltonian using a single exchange coupling of $J = 320\ \mathrm{cm}^{-1}$ and $\Delta = 750\ \mathrm{cm}^{-1}$.

The pattern can be analyzed by assuming an isotropic coupling J between either Co center and the radical bridge. Due to the inversion symmetry of the molecule, the same exchange coupling can be assumed for both centers. If we

only include the lowest quartet and neglect a direct coupling of the Co centers, the spin Hamiltonian reads

$$\hat{H}_J = J(\hat{\mathbf{S}}_1 \cdot \hat{\mathbf{S}}_\mathrm{b} + \hat{\mathbf{S}}_2 \cdot \hat{\mathbf{S}}_\mathrm{b}) \tag{S3}$$

where $\hat{\mathbf{S}}_i$ are the spin operators associated with either Co center and $\hat{\mathbf{S}}_\mathrm{b}$ refers to the bridge. The final states can be expanded in the basis $|\frac{3}{2}, M_1; \frac{3}{2}, M_2; \frac{1}{2}, M_\mathrm{b}\rangle$, which may be spin-adapted to give one octet, two sextet, two quartet, and two doublet states (32 states in total, including their multiplicities). However, because the exchange splitting turns out to be large in comparison to the splitting between the lowest two quartet states on each center (we find $J = 320$ cm^{-1}), we have to also consider coupling of the ions in their excited states to the radical bridge. We consider this extension of Eq. S3,

$$\hat{H}_{J,2\mathrm{Q}} = \sum_{k_1=0}^{1} \sum_{k_2=0}^{1} |k_1, k_2\rangle \left[(k_1 + k_2)\Delta + J(\hat{\mathbf{S}}_1 \cdot \hat{\mathbf{S}}_\mathrm{b} + \hat{\mathbf{S}}_2 \cdot \hat{\mathbf{S}}_\mathrm{b}) \right] \langle k_1, k_2| \,, \tag{S4}$$

where we assume that each quartet state (enumerated by k_1 and k_2) couples with the same exchange coupling J to the bridge. The two states on one center are again separated by an energy Δ. Note that any coupling between the quartets on one center will be mediated by spin-orbital coupling, which we will introduce in the next step (see below). The new basis states are $|k_1, k_2; \frac{3}{2}, M_1; \frac{3}{2}, M_2; \frac{1}{2}, M_\mathrm{b}\rangle$, which gives rise to 4 octet, 8 sextet, 8 quartet and 8 doublet states (128 states in total, including their multiplicities). As can be seen in Fig. S31, this simple model rather satisfactorily explains the overall pattern seen in the ab initio computations. We find four intertwined spin ladders, with origins at energies of 0, Δ (doubly degenerate) and 2Δ. There are some deviations from this model, in particular for the higher octet states, which appear at lower energies than expected from the spin-ladder model, but otherwise the model works very well. This is particularly true for the energetically lowest states that will be most important in the experiments discussed in this work.

S6.4 Influence of zero-field splitting and exchange coupling

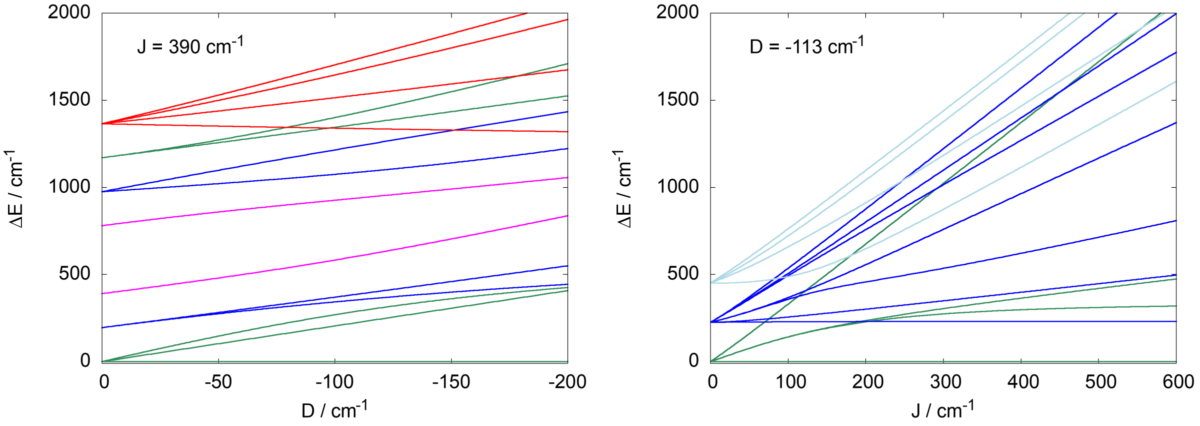

Figure S32. Eigenvalue spectrum (referenced to lowest state) of the spin-Hamiltonian 'model d1' as discussed in the main text, as a function of varying parameters. Left: Variation of the axial zero-field splitting parameter (doublet: magenta, quartet: blue, sextet: green, octet: red). Right: variation of the exchange coupling.

Figure S32 illustrates the influence of zero-field splitting and exchange coupling on the excitation spectrum of the model spin-Hamiltonian for **2**. By varying D, the transition from the pure Heisenberg spin ladders ($D = 0$) to the settings in the actual system ($D \approx -113$ cm^{-1}) can be followed. The lowest multiplets remain rather distinct, with some coupling of the quartet and the sextet $3/2$ and $1/2$ components. Fixing D to the approximate experimental value and varying J shows in particular the saturation of the first excited state at a value given by the zero-field splitting. For $J \to 0$ cm^{-1} the states collapse into four levels given by the M components of the (then isolated) quartet states of the two ions: (±3/2,±3/2), (±3/2,±1/2) and (±1/2,±3/2) (degenerate), and (±1/2,±1/2). Due to the M component of the bridge, the three levels at $J = 0$ cm^{-1} are fourfold, eightfold and fourfold degenerate (note that when the degeneracy of the Kramers doublets is included, the degeneracy is actually twice as large; however, the Kramers degeneracy is never lifted in the

present simulations, as the magnetic field remains zero).

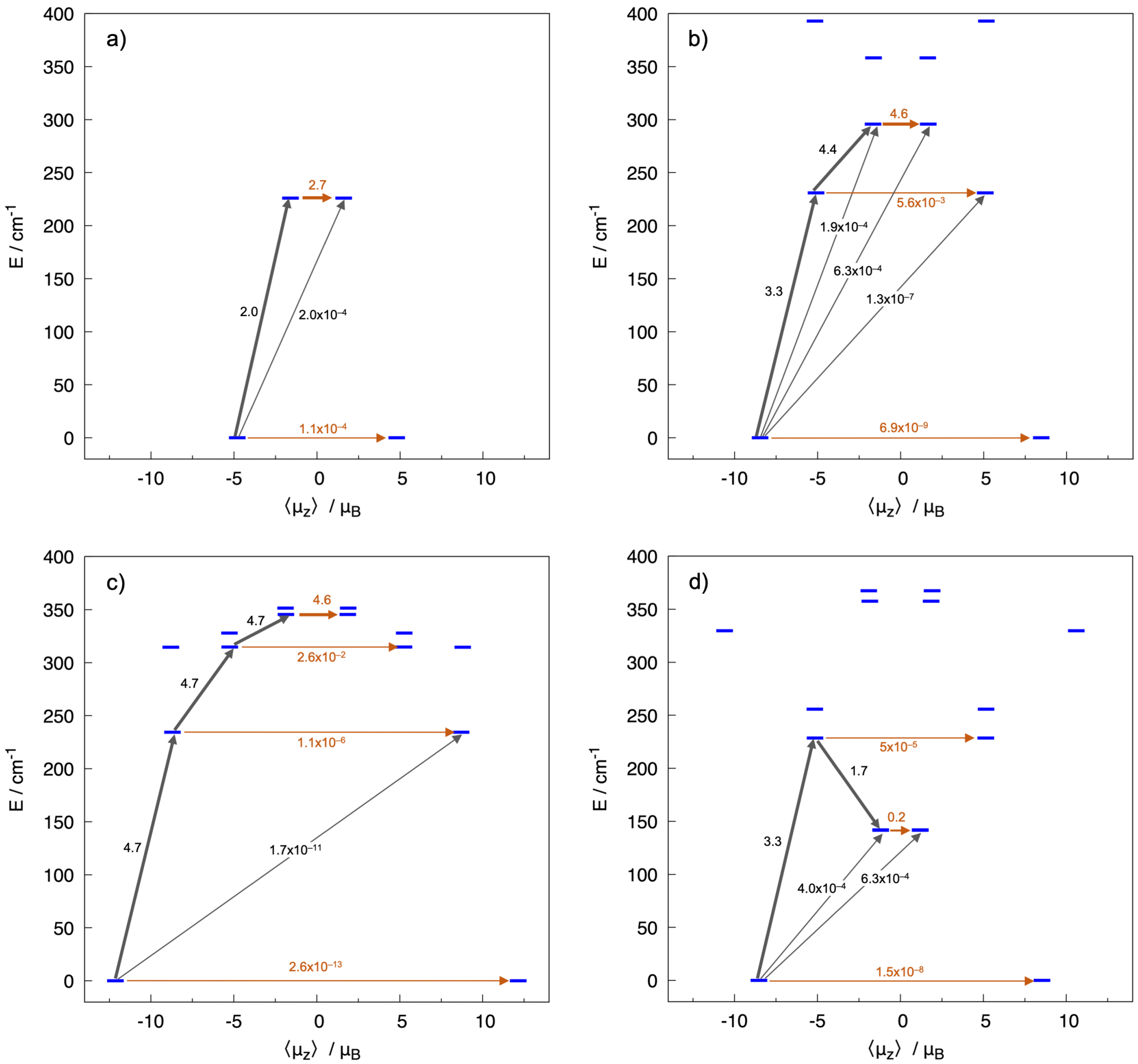

Figure S33. Simulated energy levels, expectation value of magnetic moment $\langle \mu_Z \rangle$ (in multiples of μ_B), and magnetic-field induced couplings (computed as $1/3\sum_{i\in\{x,y,z\}} |\langle I|\hat{\mu}_i|J\rangle|^2$ from the transition moments between two states I and J; in multiples of μ_B^2) for four settings: a) Mononuclear compound ($D = -113$ cm^{-1}, $E = 0.5$ cm^{-1}, $g_{||} = 3.2$, $g_\perp = 2.0$); b) radical-bridged dinuclear compound (some parameters as before, in addition $J = 390$ cm^{-1}, $g_b = 2.0$); c) (hypothetical) radical-bridged linear trinuclear compound (same parameters as dinuclear compound); d) radical-bridged dinuclear compound with reduced exchange coupling ($J = 100$ cm^{-1}; note that there are actually 2 doublets at ≈ 145 cm^{-1}, only one of them couples to the states as indicated in the graphic). The spin Hamiltonians from eqs. (1) and (2) in the main text and their obvious generalization to the linear trinuclear case are used.

The relevance of the exchange coupling for the stability of the magnetization can be illustrated by the four additional simulations shown in Figure S33. One of the mechanisms leading to quantum tunnelling of the magnetization is the interaction with stray fields from the environment, that can mix states through their couplings via the magnetic moment operator ($\hat{\mu}_i$) (where i runs over the Cartesian directions). A small mixing of the ground state Kramers doublet is, for instance, induced by a rhombic distortion E of the zero-field splitting tensor. While we have ignored this effect in most of the analysis, the ab initio computations show that there is a small rhombic component of $E = 0.5$ cm^{-1}, which gives

rise to a coupling of $10^{-4}\mu_B^2$ for the ground state doublet of the mononuclear compound. Interestingly, this value is strongly reduced to $7 \times 10^{-9}\mu_B^2$ for the exchange-coupled dinuclear compound, which demonstrates the effectiveness of the radical coupling approach pursued in this work. For a hypothetical linear trimer (with two radical bridges coupling three cobalt centers), the coupling is even further suppressed to $3 \times 10^{-13}\mu_B^2$. Strong couplings exist only to pseudospin states with $\Delta M = \pm 1$. Coupling to spin-ladder components that differ by $\Delta S = \pm 1$ are strongly suppressed by parity.

In addition, we can also show that a strong exchange coupling with $J > D$ is essential. For a much smaller coupling, e.g. $J = 100$ cm^{-1} in Figure S33 d), low-lying states emerge (see also Figure S32), which give rise to a much reduced magnetization barrier and an increase of the coupling for the ground state doublet.

S7 Supplementary Note 7 - Spin-phonon coupling simulations

S7.1 Model simulations for the mononuclear case

Fig. S34 shows the results of simulations where only a single phonon is coupled to a spin Hamiltonian. Coupling to the derivative of the axial component D does not induce perturbation of the phonon transition, while coupling to the transversal component E or any rotation of the tensor (D_{xz}, D_{yz}, D_{xy}) leads to a characteristic shift of the phonon band. We note that the effect of coupling to D_{xz} and D_{yz} leads to the same result, this is also true for the pair E and D_{xy}.

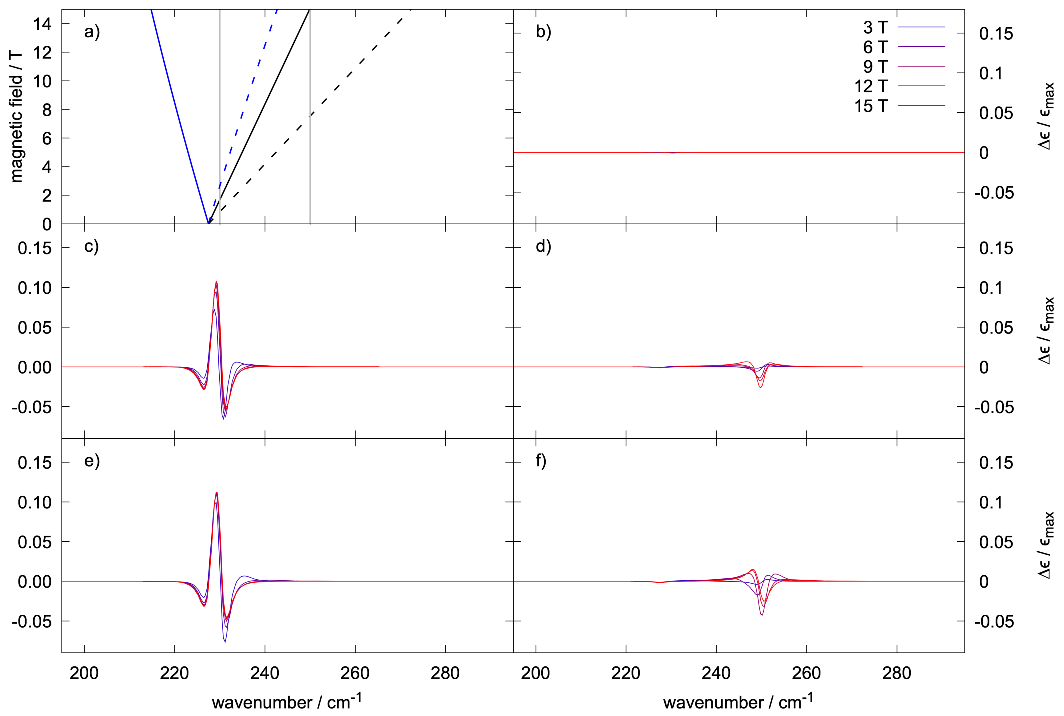

Figure S34. Interaction of a spin-3/2 system ($D = -113.75$ cm^{-1}) with a single phonon (either at 230 cm^{-1} or at 250 cm^{-1}). No spatial polarization of the phonon is assumed. a) Zeeman plot of the $M = -\frac{3}{2} \rightarrow M = -\frac{1}{2}$ (solid) and $M = -\frac{3}{2} \rightarrow M = +\frac{1}{2}$ (dashed) transitions for a magnetic field along the main magnetic axis (z-axis, black) or perpendicular to it (blue); b) coupling to a phonon at 230 cm^{-1} via the derivative $D^{(a)}$; c) coupling to a phonon at 230 cm^{-1} via $D_{xz}^{(a)}$ d) the same but with the phonon placed at 250 cm^{-1}; e) coupling to a phonon at 230 cm^{-1} via $E^{(a)}$; f) the same but with the phonon placed at 250 cm^{-1}.

S7.2 Numerical determination of spin-phonon coupling strength

Detailed values for derivatives of the ZFS tensor components (projected to normal modes of the molecular anion) are given in the Supplementary Data 2. A visualization of the findings is given in Fig. S35 and Fig. S36.

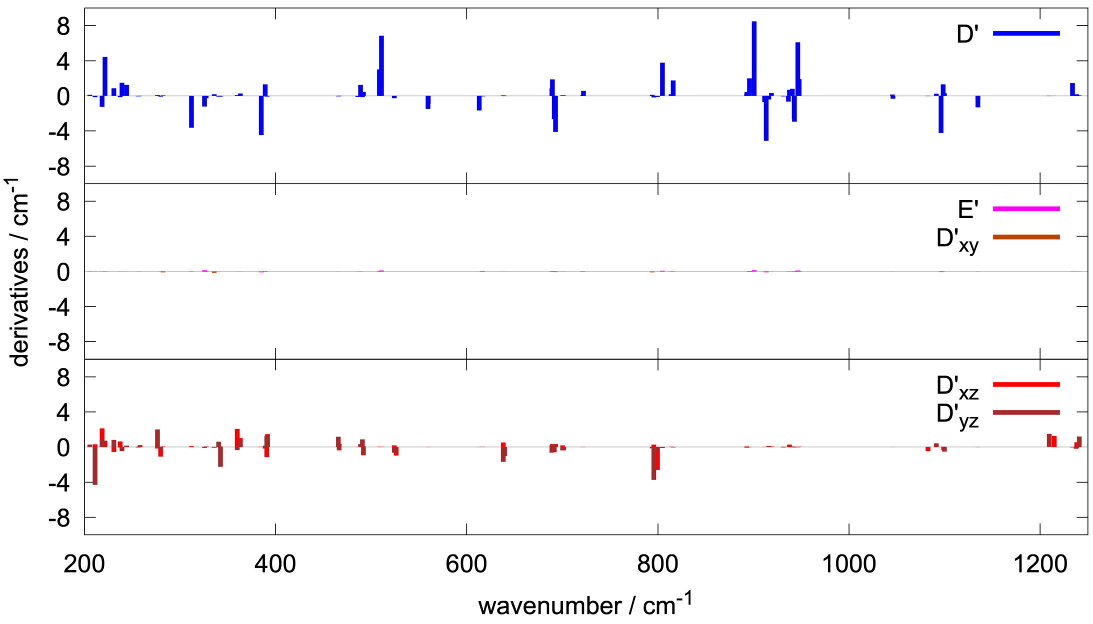

Figure S35. Visualization of the sizes of ZFS tensor component derivatives projected to normal modes for compound **1**.

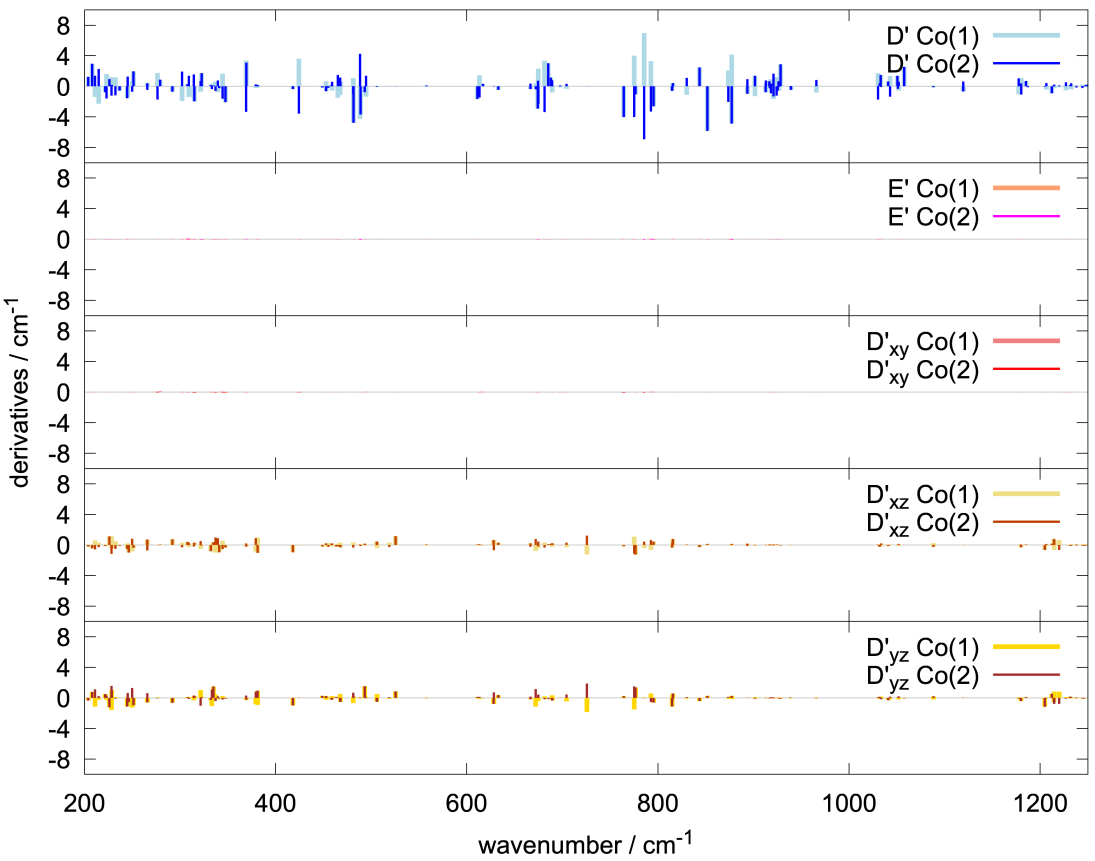

Figure S36. Visualization of the sizes of ZFS tensor component derivatives for each of the Co centers projected to normal modes for compound **2**.

S7.3 Additional simulations for the mononuclear case

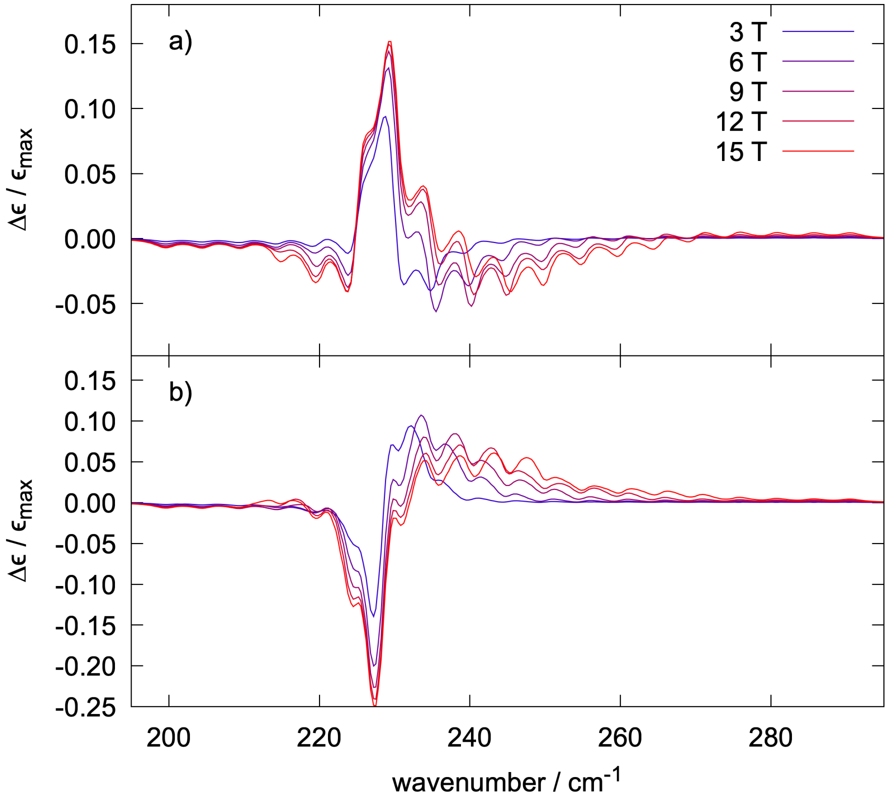

Figure S37. Simulated difference spectra (relative to zero field) of a model system consisting of a spin transition at 227.5 cm^{-1} coupled to a grid of equally spaced phonons (see main text for details). a) Simulated spectrum for coupling via $D_{xz}^{(a)}$ to the full array of modes and assuming a vanishing magnetic transition. b) Simulated spectrum if a strong magnetic transition is assumed.

S7.4 Consideration of phonon polarization

Fig. S38 shows that consideration of the polarization of the phonon (assumed as IR active) does not lead to significant differences in the overall appearance of the difference spectra.

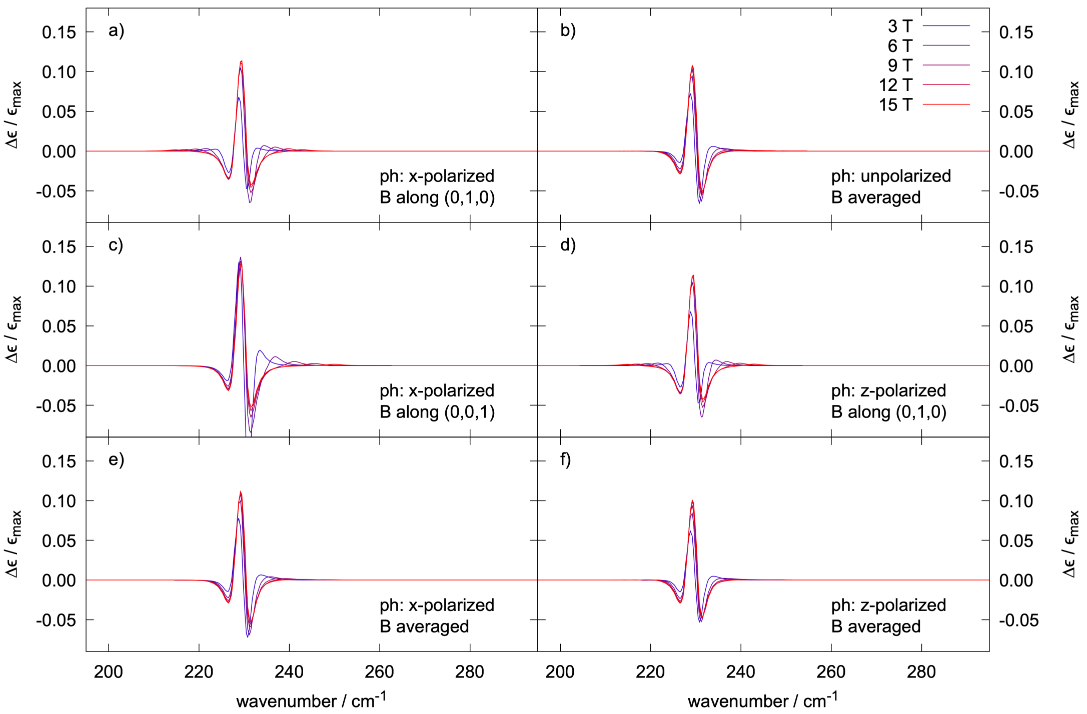

Figure S38. Test of the influence of phonon-polarization on the difference spectrum. The incident radiation is assumed to be directed along the magnetic field. When the direction of the incident radiation and the polarization of the phonon coincide, the signal vanished for all field strengths (these graphs are just constant zero lines and thus not shown). For z-polarized phonons the resulting spectrum for x and y direction is identical, therefore only the x direction is shown in panel d). The averaged spectra have been integrated by a Lebedev quadrature of order 15. The effect of different phonon-polarizations on the spectrum is shown either for distinct field directions (a),c),d), directions are highlighted in the panel) or for averaged fields (b),e),f)).

S7.5 Simulations for the dinuclear case

For these simulations, we used $D = -113\ \mathrm{cm}^{-1}$ and $J = 390\ \mathrm{cm}^{-1}$ for the spin system. The resulting states (up to $450\ \mathrm{cm}^{-1}$) as shown in the Zeeman plot in Fig. S39 (a). The first two excited spin states are the $M = \pm\frac{3}{2}$ and $M = \pm\frac{1}{2}$ components of the sextet, followed by the $M = \pm\frac{1}{2}$ and $M = \pm\frac{3}{2}$ components of the quartet state. In particular the transitions to the $M = \pm 3/2$ states show a dependency on the applied magnetic field that markedly differs from the mononuclear case, in particular for perpendicular fields. We place phonon modes at $\tilde{\nu} \in \{220, 230, 235, 240, 220, 230, 235, 240, 250, 260, 280, 300, 320, 340, 360, 370, 380, 390, 395, 400, 410, 420, 430, 440\}\ \mathrm{cm}^{-1}$ to probe the coupling with the four spin transitions that are expected in the range from 200 to $450\ \mathrm{cm}^{-1}$.

For the simulations of the spin-phonon coupling, we have to make assumptions concerning the relative phase of the spin-phonon coupling matrix elements (e.g. $D^{(a)}_{xz,1}$ vs. $D^{(a)}_{xz,2}$). The symmetry of the system implies that these matrix elements should be equal in absolute value, but may possess equal or opposite signs. The sign relation is given by the inversion symmetry of the system and the Cartesian components of the derivatives have the relation $dD_{ij,1}/dq_1 = -dD_{ij,2}/dq_2$, where q_1 and q_2 are the coordinates of two symmetry equivalent atoms in the molecule. For the normal-mode projected derivatives $D^{(a)}_{ij}$, the parity of the vibrational wavefunction is decisive. We note that also the IR intensities of the phonons will be determined by this symmetry and phonons that are symmetric with respect to the inversion center are symmetry forbidden.

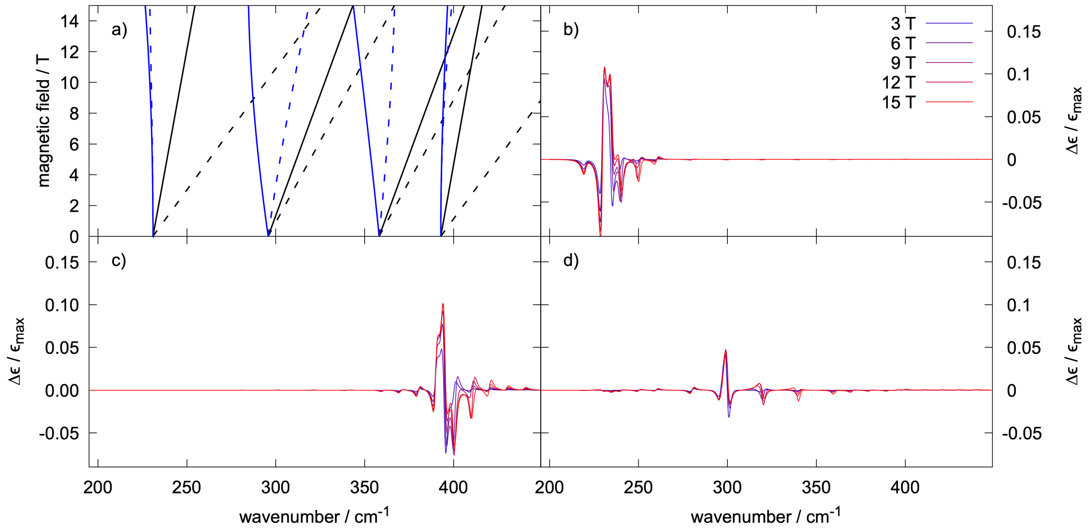

Figure S39. Simulated magnetic field dependence of two spins $S = \frac{3}{2}$ coupled to a radical bridge. a) Zeeman plot of the transitions from the $M = -\frac{5}{2}$ state for magnetic field along main axis (black) and orthogonal to it (blue); b) coupling to a set of phonons (via $D^{(a)}_{xz,c}$) and assuming even parity of the coupling matrix elements; c) the same, assuming odd parity; (d) coupling via $E^{(a)}_c$ (even parity assumed);

In a first simulation, we assumed that the coupling matrix elements (only the elements $D^{(a)}_{xz,1} = D^{(a)}_{xz,2} = 0.5/\sqrt{2}\,\mathrm{cm}^{-1}$ are considered here) are equal for all vibrational modes and have even parity. We see in Fig. S39 (b) that then only strong signals are found for the coupling to the transitions into the lowest excited spin state. The transitions into the $M = \pm\frac{1}{2}$ component of the sextet lead to a nearly vanishing signal (as $D^{(a)}_{xz}$ generally only couples to $\Delta M = \pm 1$ transitions) and the transitions into the components of the quartet vanish completely. This is because the sextet and the quartet states have themselves opposite parities, leading to an exact cancellation of the spin–phonon coupling.

Exactly the opposite can be observed when we assume $D^{(a)}_{xz,1} = -D^{(a)}_{xz,2}$, see Fig. S39 (c). In this case the transitions into the sextet components are suppressed and the coupling to the quartet state is visible. Again, only the $-\frac{5}{2} \to -\frac{3}{2}$ transition couples strongly, as we only consider $D^{(a)}_{xz}$ coupling.

We can also simulate, assuming coupling via $E^{(a)}_1 = E^{(a)}_2 = 0.5/\sqrt{2}\,\mathrm{cm}^{-1}$; the result is shown in Fig. S39 (d). Unlike for the mono-nuclear case, there is no strong signal for the lowest spin transition as the E component neither couples to the $-\frac{5}{2} \to -\frac{3}{2}$ transition ($\Delta M = 1$) nor to the $-\frac{5}{2} \to +\frac{3}{2}$ transition($\Delta M = 4$). Instead, there is a signal induced by the transition into the $-\frac{1}{2}$ component of the sextet.

In practice, the system will have both symmetric and antisymmetric vibrations, in many cases they will come in pairs. As outlined above, those vibrations that couple strongly belong to modes with vanishing IR intensity. Our numerical experiments clearly show that setting the intensity of a vibration to zero effectively eliminates the signal also from the difference spectrum.

References

1. Rechkemmer, Y. *et al.* A four-coordinate cobalt(II) single-ion magnet with coercivity and a very high energy barrier. *Nat. Commun.* **7**, 10467, DOI: 10.1038/ncomms10467 (2016).

2. Albold, U., Bamberger, H., Hallmen, P. P., Slageren, J. v. & Sarkar, B. Strong exchange couplings drastically slow down magnetization relaxation in an air-stable cobalt(ii)-radical single-molecule magnet (smm). *Angew. Chem. Int. Ed.* **58**, 9802–9806, DOI: 10.1002/anie.201904645 (2019).

3. Bamberger, H. *et al.* Iron(II), cobalt(II), and nickel(II) complexes of bis(sulfonamido)benzenes: Redox properties, large zero-field splittings, and single-ion magnets. *Inorg. Chem.* **60**, 2953–2963, DOI: 10.1021/acs.inorgchem.0c02949 (2021).
