# Supplementary material for: Electronic structure of mononuclear and radical-bridged dinuclear cobalt(II) single-molecule magnets
Source: Nat Commun. 2025 Mar 4;16:2157. doi: 10.1038/s41467-025-57210-0 (PMC11880546; doi:10.1038/s41467-025-57210-0)
Supplement: Supplementary file 2 — Description of Additional Supplementary Files [file 41467_2025_57210_MOESM2_ESM.pdf]

Description of Additional Supplementary Files

File name: Supplementary Data 1
Description: Calculated geometries.

File name: Supplementary Data 2
Description: Computational results.
